# Predictable patterns of trait mismatches between interacting plants and insects

**DOI:** 10.1186/1471-2148-10-204

**Published:** 2010-07-07

**Authors:** Bruce Anderson, John S Terblanche, Allan G Ellis

**Affiliations:** 1 Plant Animal Interactions, Botany and Zoology Department, Stellenbosch University, Private Bag X1, Matieland, 7602, South Africa; 2 Department of Conservation Ecology & Entomology, Faculty of AgriSciences, Stellenbosch University, Private Bag X1, Matieland, 7602, South Africa

## Abstract

**Background:**

There are few predictions about the directionality or extent of morphological trait (mis)matches between interacting organisms. We review and analyse studies on morphological trait complementarity (e.g. floral tube length versus insect mouthpart length) at the population and species level.

**Results:**

Plants have consistently more exaggerated morphological traits than insects at high trait magnitudes and in some cases less exaggerated traits than insects at smaller trait magnitudes. This result held at the population level, as well as for phylogenetically adjusted analyses at the species-level and for both pollination and host-parasite interactions, perhaps suggesting a general pattern. Across communities, the degree of trait mismatch between one specialist plant and its more generalized pollinator was related to the level of pollinator specialization at each site; the observed pattern supports the "life-dinner principle" of selection acting more strongly on species with more at stake in the interaction. Similarly, plant mating system also affected the degree of trait correspondence because selfing reduces the reliance on pollinators and is analogous to pollination generalization.

**Conclusions:**

Our analyses suggest that there are predictable "winners" and "losers" of evolutionary arms races and the results of this study highlight the fact that breeding system and the degree of specialization can influence the outcome.

## Background

"...a flower and a bee might slowly become, either simultaneously or one after the other modified and adapted in the most perfect manner to each other..." Darwin [1]

Using similar logic to the quote above, Darwin [2] was able to make his bold prediction that the Madagascan star orchid, *Angraecum sesquipedale*, with a spur length of 30 cm [see [3]] must be pollinated by a moth with a tongue of equally outrageous proportions. Only after his death, some 40 years later, was Darwin vindicated when a suitable candidate was found...a moth, *Xanthopan morganii praedicta*, with a tongue of 22 cm in length [4]. Not only did Darwin predict that there is strong selection on floral tubes to exceed pollinator tongues, but that there is also strong natural selection for pollinator tongues to exceed floral tubes in order to access all the nectar within the flowers of a plant population. In consequence, this theory specifically implies that a coevolutionary race [*sensu *[5]] should occur and that the length of tongues and tubes should match closely when one (or more) partner is dependent on the other [3].

One possible outcome of this prediction is that if adaptive traits of interacting organisms are geographically variable at the species or population level, then these complementary traits may be closely correlated across the distribution range of the interaction [e.g. [6]]. Indeed, in plant-pollinator as well as host-parasite systems where there seems to be strong geographic variation in adaptive traits, highly significant positive correlations have usually been found [e.g. [7,6,12]; also see [13] for predator-prey adaptations). For example, the camellia weevil (*Curculio camelliae*) studied by Toju and Sota [10,14] uses its elongated rostrum, which may reach several times the length of its body, to excavate a hole through the thickened pericarp of its host-plant, the Japanese camellia (*Camellia japonica*). The weevil's reproductive success depends upon it being able to excavate a hole deep enough that it can lay its eggs close to the seeds of its host-plant which lie deep within the defensive pericarp. The length of the weevil's rostrum is geographically variable but in each population it is closely matched with the pericarp thickness of the fruit so that the morphological traits of the plant and insect are highly correlated across geographic locations. This kind of trait covariation should not only be confined to coevolutionary scenarios but should also include unilaterally evolved systems as well [e.g. [15-17]]. Unilateral evolution refers to an evolutionary scenario whereby one organism adapts to the morphology of a second organism but not *vice versa *[*sensu *[17], c.f. coevolution, [18]]. For example, plants may adapt to novel pollinators after range expansion, but the pollinators need not necessarily adapt to the new flowers if they already have other abundant nectar sources to feed on [15] Similarly, although there is strong selection for rewardless Batesian mimic orchids to closely track pollinator traits, pollinator morphology is not driven in any way by the orchids themselves [e.g. [16]].

**Figure 1:**
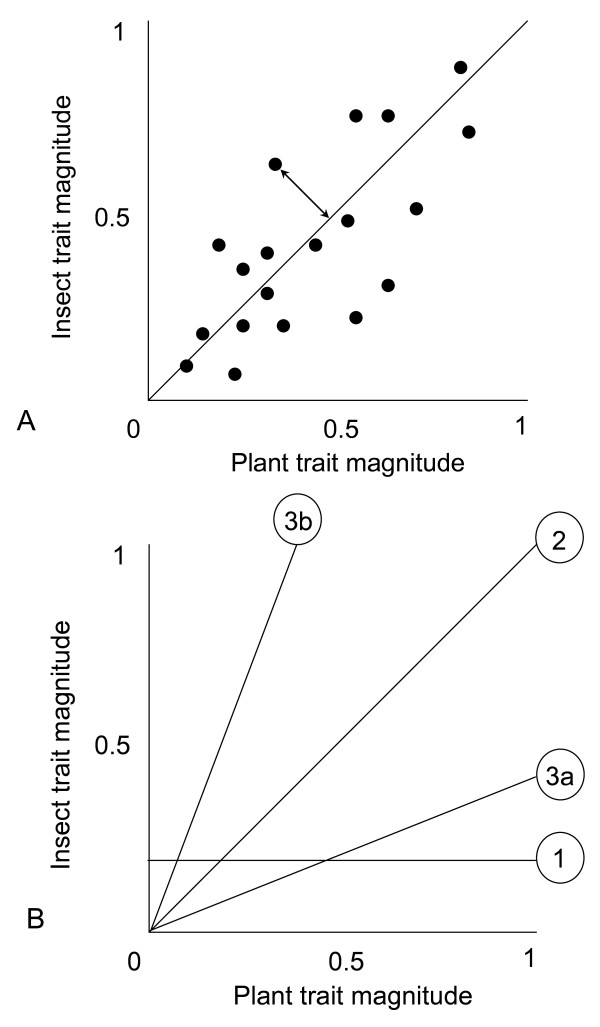
**Hypothetical outcomes of trait-matching studies**. Figure 1A. Geographic variability in the strengths of directional selection may weaken a regression (i.e. increase variability) by increasing the frequency and magnitude of trait mismatches (where a trait mismatch is indicated by a double headed arrow). However, this may not affect the slope of the relationship which indicates the predictability of the direction of the trait mismatches in relation to trait magnitude. Figure 1B. The slope of the trait regression relationship may reveal one of three possible scenarios: Slope 1, where insect and plant traits are not matched, slope 2, where trait matching scales with trait magnitude to produce a slope of 1 and slopes 3a and b where there is a consistent and predictable mismatch of traits where the mismatch is contingent on trait magnitude. For example, in slope 3a the plants have predictably longer traits than the insects and the mismatch between the taxa should become greater with trait magnitude.

Therefore, one possible, though perhaps simplistic expectation from these interactions is perfect matching between the morphological traits of interacting species pairs, but in reality mismatches are commonplace. For example, even Darwin's famous hawkmoth, which is frequently cited as textbook example of close trait-matching [e.g. [19]], has a proboscis which is considerably shorter than the nectar spur of *Angraecum sesquipedale. *In addition, Nilsson [3] observed that several studies report corolla tubes which were longer than the tongues of their pollinators. Although trait mismatches may seem counter-intuitive, the geographic mosaic theory of coevolution predicts that they should be common [20-22]. One potential reason for trait mismatches is that geographic differences in community structure can alter the strength of selection imposed by one species upon another, and *vice versa*, resulting in putatively balanced armaments in some populations but imbalanced armaments in others. Although geographic variation in the strength of directional selection may result in trait mismatches, which might weaken the correlation between co-varying traits across populations, this mechanism need not result in predictable mismatches in a particular direction across coevolving species pairs (Figure 1A). Trait mismatches in a geographic mosaic need not influence the form of the relationship between interacting phenotypes, an important component of trait matching which is less frequently reported and less well-understood than the significance of the relationship itself.

In this paper we primarily explore the influence of trait magnitude on the scale and direction of trait mismatches (or armament imbalances) between interacting plants and insects (i.e. the slope of the regression), and in particular ask whether the patterns of mismatch are predictable. Ecological and evolutionary theory make no singular prediction about the regression slope, but one possibility, under perfect phenotypic matching of coadapted traits, is that the match should be invariant irrespective of trait magnitude i.e. the slope should equal one. Counter to this prediction, the data in some studies appear to have slopes that differ significantly from one [e.g. [13,10]], suggesting that the direction and extent of trait mismatches is dependent on other factors, such as trait magnitudes. For example, in the camellia-weevil system described above, the weevil rostrum is consistently longer than the thickness of the fruit pericarp in most populations, but the phenotypic mismatch is small at high trait magnitudes and large when traits are small [10]. Importantly, deviation from 1:1 phenotypic matching does not necessarily imply functional mismatches, as has been neatly demonstrated by Toju and Sota [10]. They showed experimentally that a functionally perfect phenotypic match (i.e. the fitness of the two interacting species is balanced) occurs when weevil rostrums are 1.74 times longer than fruit pericarps. Thus, from a functional perspective, the imbalance in trait mismatch with respect to trait magnitude is greater than suggested by the phenotypic relationship alone, with plants gaining the functional upper-hand at large trait magnitudes despite close trait matching. Other than the camellia-weevil system, we know of no other studies which have determined the expected phenotypic relationships under perfect functional morphological trait matching [but see [23] for toxin production and resistance]. However, numerous studies have investigated the observed patterns of phenotypic matching between interacting plants and insects and here we focus on the predictability of the influence of trait magnitudes on the scale and direction of trait mismatch across species pairs.

Asymmetric trait matching between interacting species within a community may be expected if the strength of directional selection operating on the traits of the interacting species are different. This idea is exemplified in the controversial [24] "life-dinner" principle proposed by Dawkins and Krebs [25] where prey are thought to have stronger selective pressures acting on them than predators, because the consequence of being captured is greater than the consequence of a predator not catching the occasional prey item. As a result, it is thought that arms races are led by victims if they have more to lose than the exploiters. Brodie [26] extended this principle, hypothesizing that when a specialist interacts with a generalist, then the selection pressure on complementary traits will be greater on the specialist and so the specialist should 'lead the race.' Recent advances in understanding of the structure of networks of interacting plants and animals provide some support for the prevalence of asymmetrical specialization: Interaction networks have consistently been shown to exhibit a strongly nested structure whereby specialists interact with a subset of the species with which generalists interact [27-29]. In addition, pairs of interacting species within networks tend to exhibit asymmetry in their degree of dependence on the interaction [30,31]. Thus, asymmetry in the degree of specialization between interacting species is the norm and interactions between specialized species are rare [29]. However, in order for asymmetries in specialization to lead to consistent patterns of trait mismatches across multiple interacting species pairs, either the plants or the animals would need to be consistently more specialized. Most traditional studies on pollination guilds, syndromes and/or specialization-generalization stress that plants are typically more specialized [e.g. [32]]. However, recent network studies suggest that this is not the case - plants and animals in pollination and seed dispersal networks exhibit a similar range of specialization with plants on average more generalized than insects [29]. Thus, although asymmetry in specialization could determine trait mismatches because functionally specialized species should experience stronger selection to match (or exceed) the morphology of their interaction partner than functionally generalist species, the predictability of mismatches will depend on the consistency of specialization asymmetry across species pairs. In a similar way to generalization, plants capable of autonomous autogamy may also experience relaxed selection on their traits to match (or exceed) the traits of their pollinators because they can achieve substantial female fitness in the absence of successful pollen transfer. Consequently, plants capable of autonomous selfing may be less influenced by the evolutionary arms race with their pollinators than plants whose fitness is completely dependent on them.

In this manuscript we compile a database from the published literature, and some previously unpublished data, and review the results of specialized plant-insect relationships from the pollination and host-parasite literature. Specifically, we ask if the morphologies of interacting plants and insects are frequently mismatched, and if there is predictable directionality to mismatches when they occur. We consider that at least three possible scenarios might exist (see Figure 1): Firstly, plant and insect traits may not be matched, suggesting that the selection for trait matching is not strong enough to overcome factors that constrain the evolution of these traits. Secondly, relationships between plant and insect traits may exhibit slopes of one, suggesting that the scale and direction of mismatch is independent of trait magnitude. Note that traits are only morphologically matched if the intercept of the line passes through zero and if the slope is one (although functional matching might entail an intercept different to zero, as discussed above). Finally, although insect and plant traits may covary geographically, regression slopes may deviate significantly from one, suggesting imbalance in the extent of morphological trait matching with respect to trait magnitude. Importantly, theories such as the geographic mosaic theory of coevolution do not make any specific prediction about directionality of mismatches, and the present study therefore addresses this important knowledge gap. We then use the available data to explore some possible functional explanations for the predictable pattern of armament imbalance that we reveal.

## Results

### Population level analyses

**Figure 2:**
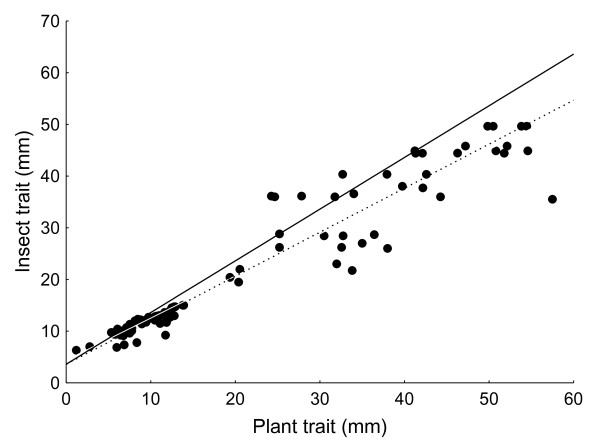
**Plant-insect trait-matching at the population level**. Scatter plot showing population means for insect and plant morphological traits from all the studies in Table 1 (excluding autonomous selfing plants). Solid line: slope of unity (intercept assumed to be the same as the value derived from the RMA regression intercept: 3.61); Stippled line: RMA derived slope through all data (slope: 0.851 ± 0.026; t_90 _= 5.7242, p < 0.0001).

Using OLS, eleven out of twelve relationships had significant positive slopes for plant versus insect traits suggesting close trait matching. Both of the parasitic relationships had slopes which were statistically shallower than one (Table 1). Using RMA, of the ten pollination relationships, eight of them were for plants which were pollinator-dependent and all of these had slopes which were <1 (3/8 significantly so, Table 1). Both antagonistic and mutualistic pollination relationships of non-selfing species consistently had slopes of less than one. In contrast, both of the autonomous selfing pollination relationships had slopes which were >1, but not statistically so (β = 1.278 ± 0.325, t_6 _= -0.856, p = 0.788 and β = 1.417 ± 0.518, t_3 _= -0.806, p > 0.2396). Furthermore if all slopes are analyzed together (mean β = 0.7729 ± 0.31916 (SD)) against an expected slope of one using a one sample t-test, then this analysis also suggests that slopes are significantly shallower than one (t_11 _= 8.3910, p < 0.00001). Excluding autonomous selfers (which probably have putatively relaxed selective pressures on floral traits, see discussion) from this analysis only serves to reinforce the pattern of the slope being less than one (mean β = 0.6581 ± 0.1880 (SD), t_9 _= 11.0670, p < 0.00001). Similar results were obtained if the axes were reversed (results not shown) or if the slopes were analysed using ordinary least-squares (OLS) regression (Additional file 1). If the data from all population studies are combined as a single RMA regression (excluding autonomous selfers), the slope (β = 0.8514 ± 0.0280) of the relationship is significantly shallower than one (t_90 _= 5.724, P < 0.0001, Tabe 1, Figure 2). If only antagonistic/parasitic species are grouped together, the RMA slope of the relationship is significantly less than one (β = 0.8104 ± 0.053, t_29 _= 3.5720, p < 0.001), and when all mutualistic (non-selfing) species are grouped together, (β = 0.8625 ± 0.028, t_59 _= 4.9585, p < 0.00001). Thus, in general, whether analyzing populations within species (Table 1) or pooling populations across species (Figure 2), imbalances in trait matching with respect to trait magnitude appear to be common and consistent, favouring the plants most when trait magnitudes are large.

**Table 1:** Summary of reduced major axis (RMA) regression results for tests of morphological symmetry in adaptive traits within and among reported study systems.

Insect	Plant	Reference	Co-evolved/ Unilateral	Breeding system	Interaction	Slope ± SE	Intercept ± SE	R^2^	*t*reg, Preg	*t*	Pslope^†^	DF
*Curculio camelliae *(C)	*Camellia japonica*	[10]	C	NA	Parasitic	0.6878 ± 0.058	5.043 ± 0.591	0.908	-11.85, <0.0001	5.383	**<0.000061**	13
*Jadera haematoloma *(H)	Sapindaceae	[58]	?	NA	Parasitic	0.2654 ± 0.0503	5.778 ± 0.356	0.857	-5.276, <0.0031	14.604	**<0.000064**	4
*Tabanid *sp. (D)	*Disa draconis*	[76]	U	O	Pollination (A)	0.5598 ± 0.0875	10.779 ± 3.658	0.951	-6.398, <0.01179	5.031	**<0.01866**	2
*Rediviva neliana *(Hy)	*Diascia *spp.	[7]	C	O	Pollination (M)	0.6314 ± 0.0374	6.045 ± 0.3306	0.907	-16.88, <0.00001	9.856	**<0.000001**	19
*Rediviva neliana *(Hy)	*Diascia capsularis*	[7]	C	O	Pollination (M)	0.8471 ± 0.2002	3.308 ± 2.432	0.721	-4.231, <0.00412	0.764	0.2397	5
*Rediviva pallidula *(Hy)	*Diascia *spp.	[7]	C	O	Pollination (M)	0.4594 ± 0.0650	7.892 ± 0.618	0.920	-7.068, <0.00106	8.317	**<0.00057**	4
*Prosoeca ganglbaueri *(D)	*Disa nivea*	[16]	U	O	Pollination (A)	0.7037 ± 0.1931	12.946 ± 7.83	0.699	-3.644, <0.0109	1.534	0.0999	4
*P. ganglbaueri *(D)	*Zalusianskya microsiphon*	[6]	C	O	Pollination (M)	0.7866 ± 0.1172	6.456 ± 4.5	0.689	-6.712, <0.00001	1.821	<0.045	14
*P. ganglbaueri *(D)	*Gladiolus oppositiflorus*	[17]	?	O	Pollination (M)	0.7616 ± 0.1628	11.266 ± 6.953	0.817	-4.678, <0.0047	1.464	0.1085	4
*P. ganglbaueri *(D)	*Amaryllis *spp.	[17]	?	O	Pollination (M)	0.8777 ± 0.1892	0.6731 ± 7.757	0.861	-4.639, <0.0094	0.646	0.2822	3
*Moegisterynchus longirostris *(D)	*Lapeirousia anceps*	[32]	C	AS	Pollination (M)	1.278 ± 0.3247	-7.212 ± 18.88	0.613	-3.936, <0.0038	-0.856	0.7876	6
*M. longirostris *(D)	*Babiana tubulosa*	[32]	?	AS	Pollination (M)	1.417 ± 0.5176	-42.896 ± 40.531	0.600	-2.737, <0.0358	-0.806	0.2396	3
*ALL POPULATIONS (except AS)*						0.8622 ± 0.0226	3.621 ± 0.603	0.938	-38.15, <0.0001	6.097	**<0.00001**	73

Note that the origin of the regression was allowed to vary freely (i.e. not constrained to zero). Plant breeding system is indicated for pollination relationships with O = outcrossing and AS = Autonomous selfing, and the type of interaction is categorized as parasitic or pollination. Pollination relationships are further divided into mutualistic (M) or antagonistic (A) relationships. Preg = probability value for the t-test for regression significance, Pslope = probability value for the comparison of estimated slope against expected unity (a value of 1.0), DF = degrees of freedom. The slope for "all populations" was derived by pooling all data points within each of the studies below, and not by averaging the slopes of each relationship. Insect order is indicated by a letter in parentheses where C = Coleoptera, H = Hemiptera, D = Diptera, Hy = Hymenoptera. † Bold values are significant at p = 0.05 after False Discovery Rate correction.

#### The effect of community and functional generalization/specialization

**Figure 3:**
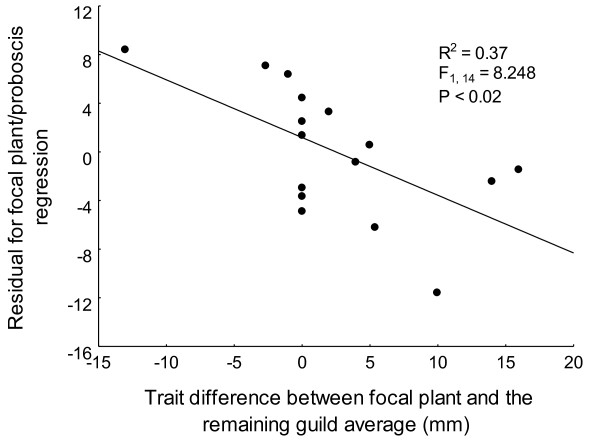
**The effect of community composition on trait-matching**. The negative OLS regression between trait mismatching and community heterogeneity. Community heterogeneity was measured as the mean difference in corolla length between a single focal plant (*Zaluzianskya microsiphon*) and other food sources found at the site. Trait mismatching was calculated from the residuals of the regression between pollinator (*Prosoeca ganglbaueri*, Y-axis) proboscis length and the corolla tube length of its most important and widespread host plant (*Zaluzianskya microsiphon*, X-axis). Negative residuals (Y-axis), indicate that fly proboscis length is shorter than predicted by the plant-proboscis regression. Negative values on the X-axis indicate *Zaluzianskya *has smaller tube lengths than the guild average.

We found that trait mismatches between the focal plant and pollinator were related to the heterogeneity of trait magnitudes within the plant community (R^2 ^= 0.37, P < 0.02, Figure 3). When other nectariferous plants within the community had nectar tube lengths which differed greatly from the focal plant, it was likely to find a large mismatch between the pollinator and the focal plant as well.

### Inter-specific analyses

Our analysis used 31 different insect species visiting a total of 115 different plant species in our dataset (a total of 137 interactions). The average number of insects per plant across the data set is 1.181 (SD: 0.387) while the average number of plants per insect is 4.419 (SD: 4.931) probably indicating a strong asymmetry in the degree of specialization. Overall, there is a significant difference between insects and plants in terms of the number of interactions (χ^2 ^= 103.45, df = 1, p < 0.0001).

**Figure 4:**
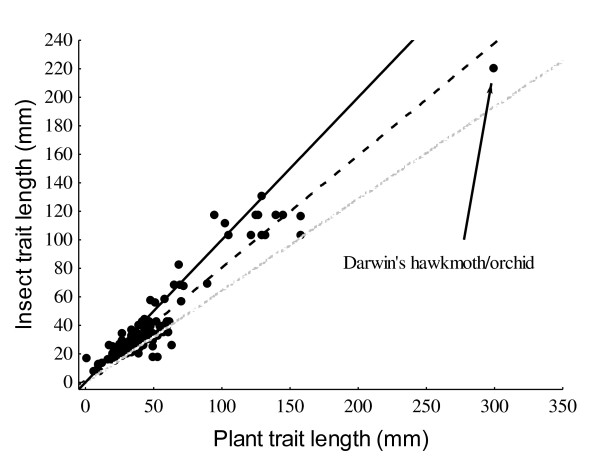
**Plant-insect trait-matching at the species level**. Scatterplot showing the interspecific relationship for morphological matching between insect and plant traits. The solid line indicates the slope of unity or symmetry, the stippled line is the conventional OLS regression analysis and the grey dotted line gives the most likely statistical model, namely the phylogenetically-adjusted model incorporating the insect phylogeny.

Before adjusting for phylogeny, the slope of the species-level regression was 0.789 ± 0.0217 (t_134 _= 36.335, R^2 ^= 0.908, p < 0.00001) which was significantly shallower than a slope of one (t^134 ^= 9.719, p < 0.00001). Results from the inter-specific PGLS analyses indicated that the phylogenetically-adjusted model which incorporated the insect phylogeny was the best model, while the probability that either the conventional or PGLS model using the plant phylogeny was correct was extremely small (Table 2). The trait matching relationship from the PGLS adjusting for the insect phylogeny yields a slope shallower (β = 0.645 ± 0.0277) than the conventional analysis and substantially less than one (t_134 _= 12.779, p < 0.00001), again suggesting trait mismatches which predictably favour the plants more at high trait magnitudes. RMA regression of species-level results produced a similar result (Table 2). Furthermore, these species level results (Figure 4) mirror the trend seen for population-level analyses (Figure 2), whether adopting a conventional, or a phylogenetically-adjusted statistical approach, and irrespective of the choice of phylogeny (plant vs. insect, Table 2).

**Table 2:** Summary of the models examined to explain variation in morphological patterns of trait matching between insect and plant species.

Phylogeny	Analysis	λ lambda	AIC	*w*_*i*_	*ΔAIC*	*Slope *± *SE*	*UCL:LCL*	*P*	*Df*
	Conventional		1031.1	<0.0001	36.82	0.783 ± 0.023		<0.0001	135
Insect	Phylogenetic	0.797	988.6	>0.999		0.645 ± 0.028	0.700: 0.590	<0.0001	
Plant	Phylogenetic	0.499	1025.4	<0.00001	42.52	0.765 ± 0.025	0.815: 0.715	<0.0001	

	RMA					0.827 ± 0.022	0.784: 0.870	<0.0001	

Each model was tested using either (1) an actual phylogenetic hypothesis for one taxon (plants or insects), assuming equal branch lengths (see Methods), or (2) a "star-shaped" phylogeny with all species equally related, equivalent to a conventional non-phylogenetic analysis or (3) using reduced major axis (RMA) regression without any phylogenetic adjustment. LCL: lower 95% confidence limit; UCL: upper 95% confidence limit. λ lambda is a measure of phylogenetic correlation (for details of how this is calculated see Halsey et al. 2006); AIC is Akaike's information criterion; *w*_i _is the Akaike weight, the probability that the model is the correct one of those tested [67]. RMA confidence limits calculated by bootstrapping (1000 bootstraps) in RMA for JAVA v. 1.21 [63].

#### The effect of plant breeding system on trait matching

**Figure 5:**
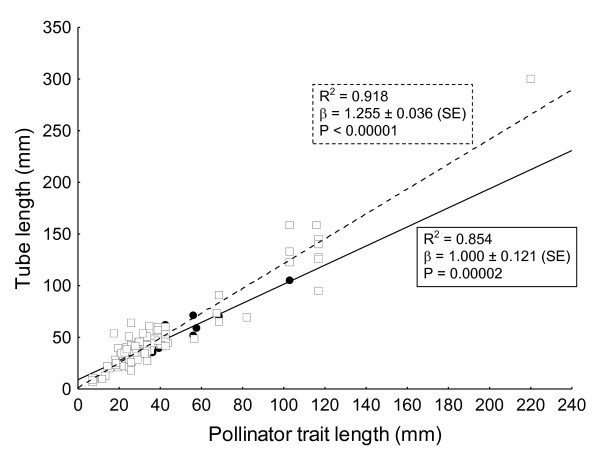
**The effect of plant breeding system on the magnitude of trait-matching slopes**. RMA regression slopes of pollinator tongue or leg morphology against the tube length of obligate insect pollinated plants (clear squares and dotted line) and plants capable of autonomous selfing (solid circles and solid line). Note that the axes have been changed in this analysis because we are testing whether tube length is dependent on plant mating system. P-values refer to tests against a slope of 0.

Using RMA, we found that the mean slope for pollinator-dependent plants was 1.255 ± 0.036 (±SD), (Figure 5). In contrast, the regression slope for autonomous selfers was 1.000 ± 0.121 (±SD), (Figure 5). Slopes were very similar using OLS (results not shown). However, there was a significant interaction effect of autogamous versus pollinator-dependent plants on the steepness of the pollinator-plant trait length slope (Wald χ^2 ^= 5.187, df = 1, p = 0.02), with the slope for pollinator-dependent plants being steeper than for autonomous selfers, meaning that pollinator-dependent plants had more exaggerated morphological traits at high values than plants which were able to self pollinate.

## Discussion

These results, from both the population- and species-level analyses, suggest that local adaptation of one species to another frequently produces strong correlations between putatively adaptive traits. However like many other studies [e.g. [10,33-39,11]], our results also show that mismatches often occur in biological systems. In particular, we provide evidence for the ubiquity of imbalances in the "armaments" of complementary traits. But, most importantly, we suggest that there may be a predictable pattern to these mismatches which is repeated at several taxonomic levels [also see [39]]. Here, matching/mismatching refers to the degree at which trait matching scales with trait size. At low trait magnitudes, traits are reasonably matched or mismatches favour the insects, whereas at high trait magnitudes, plant traits are usually larger than those of insects. To use a classic example, Darwin's hawkmoth has a proboscis which is substantially shorter than the spur length of the Madagascan star orchid (Figure 4). Our results also support Nilsson's [3] casual observation that when moths and flowers evolve long tubes and tongues, the flowers most frequently seemed to have longer floral tubes than the moth proboscides. This trend holds for the population- and species-levels irrespective of method or statistical approach used. This pattern of mismatching not only holds for multispecies, co-evolved pollination mutualisms [e.g. [8]], but also for potentially pairwise co-evolved host parasite relationships [e.g. [10,14]] and, although the unilaterally evolved studies were lacking in sample size, the trend remained the same for all of these studies too. The notable exceptions to this rule were the two population studies where plants are autonomous selfers. In addition, when the 12 autonomous selfers were analysed separately in the species-level data set, they were found to have a slope value which statistically approximated one, suggesting that plants were not gaining a morphological advantage over their pollinators at high trait magnitudes, perhaps because selfing in the absence of pollinators relaxes the selective pressure imposed by the pollinators. In contrast, plants reliant on pollinators for seed set had more exaggerated morphological traits than their insect pollinators indicating that the adaptive advantage lies with the plants. Furthermore, the difference in the steepness of these slopes was found to be significant after testing for an interaction between tube length and pollinator reliance.

### Asymmetrical specialization and predictable imbalances in trait matching

Figure 3 suggests that if an insect species forages from variable food sources, then it may be less likely to match a particular focal plant species than if it was foraging on that species alone. Thus, the strength of selection on mouthpart or floral traits may be dependent on community context [see [20,40]]. For example, insects with excessively long tongues may be selected against by having longer handling times on plant species in the community with shorter tubes than the focal species [41-43]. For this mechanism to generate the pattern we describe above (i.e. larger trait mismatches in favour of the plants at higher trait magnitudes) plants would need to be more specialized than their animal partners and the degree of asymmetry should be highest at high trait magnitudes. Several studies have found evidence for this in that long tongued insects are able to access nectar from a wider range of plant species than insects with short tongues [e.g. [44,45]]. Haber and Frankie [46] found that the proboscis length of hawkmoths was positively correlated with the number of plant species utilized so that longer tongued moths tended to be more generalized than shorter tongued moths. In contrast they found that long-tubed flowers were more specialized than short-tubed flowers as fewer pollinators were able to access the nectar. Thus, at lower trait magnitudes, insects are expected to be relatively more specialized than plants but at higher trait magnitudes, plants are expected to be relatively more specialized than insects. This type of asymmetry in specialization across trait values is likely to give rise to asymmetry in trait values if selection pressure is linked to the level of specialization in a community. In the data set used for this paper, plants were usually highly specialized and so it is more likely that levels of insect generalization are driving the asymmetry.

Contrary to current understanding of the interaction webs within which coevolution takes place, we find that published studies of trait matching generally involve interactions between specialist plants and more generalist animals (31 insect species versus 137 plant species). Why coevolutionary studies should represent a non-random subsample of interactions from many plant-animal mutualistic networks remains an intriguing and open question. However, it is possible that generalized plant - specialized insect systems seldom produce exaggerated morphological traits or produce a different set of traits and thus trait measurements are not reported in the literature. For example, traits associated with specialized pollen collecting bees may be hard to measure [e.g. [47]], particularly if specialized species are more rare. Interestingly, simulations have shown that coevolutionary complementarity (trait matching) and subsequent phenotypic convergence can generate strongly nested interaction networks i.e. asymmetric specialization [21,48-50]. Thus, coevolutionary trait matching of a focal species pair and subsequent evolutionary convergence of other taxa may generate asymmetry in the degree of specialization, which, coupled with geographic variation in the selective environment (e.g. number of converged taxa) or in the identity of the focal species pair, may generate the consistent trait mismatch in favour of the more specialized partner that we demonstrate here.

The effect of asymmetric specialization was explored further by examining mismatches between the long-tongued fly (*P. ganglbaueri*) which forages from a variable guild of plants and its widespread and primary host plant (*Z. microsiphon*) which is pollinated almost exclusively by *P. ganglbaueri*. These mismatches in complementary morphological traits were related to the variability of the nectar producing community used by the fly at each site. The focal plant (*Z. microsiphon*) had more exaggerated morphological traits than the pollinator when pollinators foraged from other plants with very short traits. The morphological traits of insects were more exaggerated than the focal plant if the background plant community consisted of species with even longer tubes. Although the relationship between mismatches and variability in the floral traits of the nectar community is very strong, some of the variation in this relationship may still be explained by the fact that the analysis did not take abundance or nectar quality of each species into account. Verification of these results using other systems and more advanced methods would add greatly to this field of biology. Nevertheless, the results from this study lend support to the idea that community context [20] and the symmetry of reciprocal specialization [life - dinner principle, [25]] can play decisive roles in patterns of trait matching and local adaptation. Furthermore these results suggest that evolutionary races can be won or lost by being a functional specialist (foraging from a consistent, single food source) or a functional generalist (foraging from a variable or diverse range of food sources).

The degree of symmetry in specialization, or more particularly dependence, seems also to be affected by plant mating system which can in turn affect trait evolution. Comparisons of pollinator-plant traits in plants with different mating systems revealed that autonomous selfers had steeper slopes than plants dependent on insects for pollination. Thus, if pollinated by the same pollinator, an autonomous selfer would have a shorter tube length than a plant species reliant on pollinators for seed set or seed siring. Autonomous selfing effectively reduces a plant's reliance on pollinators, neutralizing the asymmetries normally observed in interactions where plants are reliant on insects for seed set. Plant mating system can be viewed in the same way as specialization, with autonomous selfers being the equivalent of generalists, and so this data also lends support to the life-dinner principle [25] which suggests that specialists may outpace generalists in arms races because selection for extreme traits is greater for specialists than generalists.

### Alternative mechanisms resulting in predictable trait mismatches

Although asymmetric specialization may contribute to mismatches between pollinators and plants, it is unlikely to account for trait mismatches in the host-parasite interactions examined in this study as they appear to be reciprocally specialized. Similarly plant breeding system is unlikely to influence the pattern of mismatches in the host-parasite systems investigated because the host-plants in this study do not depend on their parasites for seed production. Thus, despite the simplistic appeal of asymmetric specialization or plant breeding systems as an explanation for the pattern we report, it is likely that other factors may also be important.

A likely alternative explanation is differences in the physiological and developmental constraints on evolution of traits in animals and plants. It may be relatively more costly for insects to move around with extremely long mouthparts or legs because it hampers foraging efficiency and contact time on plants, as well as being more costly in terms of flight energetics and simple insect body plan construction costs [e.g. [51-54]]. Alternatively, physical constraints, of e.g. nectar uptake rates relative to nectar concentration or proboscis dimensions [41,43], may become more critical at high trait values. In contrast, the production of extreme traits in plants is only associated with construction costs (although these may be expensive in some cases such as the Japanese *Camellia*, [10]). Asymmetries in physiological constraints could generate the observed pattern of consistent trait mismatches within species, and in addition, it is a mechanism which potentially scales up to generate the same pattern across species (e.g. scaling of insect resting or flying metabolic rate [54,55]). Support for this possibility is provided by the fact that we find that it is important to correct for insect phylogeny, but not plant phylogeny, in our species-level analysis, suggesting that insect mouthparts may be more constrained by phylogeny than plant flower parts. This result is similar to that of Rezende et al. [56] who found that phylogeny is significantly correlated with ecological similarity in 60% of interaction webs for animals, but only in 25% of webs for plants. In another study Rezende et al. [49] also show that phylogeny significantly influences trait distributions for animals but not for plants, lending further support to the idea that the evolution of complementary traits is more constrained in animals than in plants.

Differences in the relationship between phylogeny and ecological similarity (or phenotype) for plants and animals might also result from differences in the mobility and evolvability of each group [56]. For example, differences in the spatial scale of adaptive responses between plants and insects could result from the homogenizing effects of gene flow if insects move further than pollen or seeds. Mismatches on either end of the trait continuum could result from the constraint on evolutionary matching imposed by insect dispersal from populations with intermediate trait values. Although insects intuitively seem more mobile than plants, Morjan and Rieseberg [57] show that historical gene flow levels (as estimated from neutral molecular markers) are roughly equivalent across plants and insects, especially when only considering gene flow through seed dispersal. Importantly, substantial overlapping variation in gene flow estimates exists in both groups, contingent on species-specific life-history, dispersal and behavioral attributes. Thus, it is unlikely that the direction of gene flow mismatches between interacting plant and insect species is predictable, but should instead vary depending on the specific traits involved. Moreover, asymmetrical gene flow cannot easily explain trait mismatches across species.

Morphological mismatches may also favour plants if they consistently evolve faster than insects and stable evolutionary equilibria have not yet been reached. This could occur if plants are usually found in larger, more genetically-diverse populations than insects or if plants have faster generation times. Although the former could be true for many pollination systems, it is unlikely to be the case for most host-parasite systems [e.g. [10,57]]. In addition, insects tend to have much faster generation times than plants, with many specialized plants being long lived or perennial [59], so we consider it unlikely that differing rates of evolution is the primary explanation for these mismatches.

Hanifin et al. [39] found that garter snakes (*Thamnophis sirtalis*) evolve resistance to the neurotoxin of their newt prey (*Taricha*), and that occasionally the snakes were able to escape the coevolutionary arms race by evolving extreme resistance phenotypes. Interestingly, the salamanders never gained the evolutionary "upper hand" in this relationship and so imbalances in armaments always favoured the snakes. They proposed that the imbalances in armaments in this system were related to the genetic architecture of the complementary traits under selection. For example, it may be easier to evolve extreme resistance than it is to evolve extreme toxicity. Similarly, differences in the evolvabilty of the traits in our study may have played a role in determining winners and losers in plant-insect systems, with extreme floral traits being more easily evolved than extreme insect traits. This however was not tested in any way in our study, but remains a possible avenue for future research.

## Conclusions

In conclusion, this study demonstrates that there is a consistent and predictable asymmetry in the development of "armaments" in interacting plants and insects. These data suggest that plant morphological traits are more exaggerated than insect traits at the long end of the spectrum of traits measured, and this could have far-reaching implications for community ecology and the evolution of species interactions. Mismatches may be more pronounced at the long end of the spectrum because pollinators with exaggerated morphological traits are able to utilize a greater subset of plants than insects with less exaggerated morphological traits. Insects with exaggerated morphological traits may therefore be less dependent on a single food source than insects with less exaggerated traits, which might have implications for climate change related risks on pollinator (and other interaction) networks [see [60]]. In pollination systems, the pattern of trait mismatches was also affected by the level of dependence of plants on their pollinators. Species capable of autonomous selfing did not show the pattern of exaggerated morphological trait imbalance at higher trait magnitudes prevalent in species reliant on pollinators for fruit set, perhaps because autogamy eliminates their reliance on pollinators and thus the asymmetry in the dependence of one partner on the other. In order to better understand the functional significance of the regression slopes, it is necessary to start investigating pollination relationships in terms of their phenotypic interfaces as demonstrated in the studies of Brodie and Ridenhour [23] and Toju and Sota [10]. The phenotypic interface is an important but as yet neglected avenue for studying functional trait matching and we hope that this paper stimulates such studies in the future. Asymmetries in specialization are also not the only explanation for these patterns and are unlikely to explain the slope patterns evident in some host-parasite systems [e.g. [10]]. Consistent differences in constraints on trait evolution in insects and plants may also play roles in determining the imbalance of armaments. For example, foraging and flying may be hampered in insects with extremely long mouthparts but plants with extreme traits may not suffer similar constraints. We also hope that this paper stimulates future research on the costs and mechanisms which constrain the evolution and matching of morphological traits in plants and insects. It is evident that there is a real paucity of non-pollination studies in this data set and we believe that population level trait-matching in host-parasite systems [e.g. [10]] is extremely understudied. This is an exceptionally important area of study as it has applications for the control of pests, disease, drug resistance and biological invasions [see [39]]. As a result, understanding what determines the outcomes of interactions in model systems may have practical implications for combating disease, pests and invasions. Because there are only a small number of published studies available on morphological trait matching, we see a need for more studies which determine where and when armament imbalances occur as well as whether the directionality of armament imbalances is as consistent as suggested here. In particular, there is a need for future studies to report trait measurements in other taxa such as hymenoptera (proboscis lengths), which are absent from our data set.

## Methods

We searched the Anglophone literature for plant-animal interaction studies which reported morphometrics of matching plant and insect traits (e.g. corolla length and proboscis length; see Additional file 2). Studies which did not measure morphological variables on similar dimensions (i.e. in units of length) were excluded from the database. We only used systems where available literature suggested that one or both partners were completely specialized at either the species or population level. We divided these studies into two groups: studies on multiple populations within a species and studies where only a single data point was provided for each species.

### Population level analyses

Because not all studies investigating morphological matching between insects and plants present slopes of relationships and tests for homogeneity of slopes, we re-analyzed data compiled from previous studies with plants on the X-axis of the regression as well as with plants on the Y-axis of the regression. Reversing the axes of the regressions did not significantly affect the general analyses (i.e. which taxon wins the evolutionary race), thus for simplicity we generally present all data with insects on the Y-axis. Analyses were undertaken using reduced major axis (RMA) regression and repeated using ordinary least-squares (OLS) regression on the raw (i.e. untransformed) population level data for each study separately. Where possible, RMA was reported when we were specifically interested in comparing slope values but OLS was also reported when we were interested in whether or not there was a significant relationship between insect and plant traits [see [61]]. In all cases for a particular species complex we tested firstly, for a significant match between plant and insect traits (i.e. a significant regression) and, secondly, for imbalance in trait matching with respect to trait magnitude (i.e. deviations of the regression slope from one). To do this we tested for slope homogeneity against an expected value of one using a two-tailed t-test [[62]; p. 360] at the population level for each study separately and then also across all studies. RMA analyses were undertaken in RMA for JAVA v. 1.21 [63]. Results of OLS regression also support all our major RMA results and conclusions and suggest the effects we discuss are not simply issues of different forms of measurement error among insects and plant traits. The two autonomous selfing relationships were excluded from this analysis across studies since we were interested in controlling for this factor. These RMA and OLS analyses were repeated with only parasitic/antagonistic species and for mutualistic species alone. Table-wide false discovery rate correction was used to correct for repeated hypothesis testing [64].

The origins of the regressions were not forced through zero as the measurement of plant and insect traits may differ based on the investigators' perception of the appropriate functional trait magnitude. Consistent differences in measurement across taxa will not influence the slope of the regressions, but will influence the intercepts. One possible problem with this approach is that consistent biologically relevant variation in the intercept itself, due for example to plants and insects having different lower size constraints, might influence estimates of the slope. For example, if the intercept of an interaction always involves longer insect traits than plant traits, we might expect an initial phase (with slope ≠ 1) involving the plant "catching up" with the insect, followed by a secondary phase involving coadaptation or sequential evolution (with slope = 1). Therefore, we also tested for this possibility using breakpoint (piece-wise) regression and found no evidence for the two phase model providing a better fit or lower mean-squared error than ordinary regression (results not shown).

#### The effect of community and functional generalization/specialization

To explore the idea of asymmetrical specialization leading to trait mismatch in more depth, we used data from the studies of Anderson and Johnson [6,17]. Anderson and Johnson [6] studied geographic covariation between the corolla length of an important rewarding plant (*Zaluzianskya microsiphon*) and the tongue lengths of its sole fly pollinator (*Prosoeca ganglbaueri*). The two are thought to have coevolved at several sites and are closely matched morphometrically at most sites. However, in a subsequent study Anderson and Johnson [17] reveal a whole community of other plants which are also visited by this pollinator, although most of the other community members are not particularly widespread. At 16 different sites, *Z. microsiphon *and all other community members visited by this long proboscid fly were documented and measured. In relationships where one species is more specialized than the other (i.e. plant more specialized than pollinator), the pollinator may frequently be exposed to more variable selective pressures than the plant (i.e. a more morphologically variable food source). If the plant community plays a role in determining trait matching between a focal plant and its pollinator, then we expect poor trait matching in variable plant communities but good trait matching in less variable plant communities. Next, by pooling corolla length data from all other plants utilized by the fly (N = 20 per species), we calculated the mean corolla length of the other plants utilized by *P. ganglbaueri. *We then calculated the mean difference between *Z. microsiphon *corolla lengths and the rest of the nectariferous plants utilized by the pollinator at each site. This difference was related to the degree of mismatch at each site, measured as the residuals from the trait regression of *P. ganglbaueri *(Y axis) and *Z. microsiphon *(X-axis) across sites. Ideally, the importance of each species' corolla length as a selective agent should be weighted according to its relative abundance and nectar quality, but unfortunately we do not have these data for all of the sites and species found there.

### Inter-specific analyses

Next, using a similar approach to the population-level analysis, and assuming that measurement error and observer bias is similar across all taxa and species, we investigated the patterns of trait matching across multiple interacting species pairs. To adjust for the statistical non-independence of species data or possible clade effects, relationships between plant and insect morphology were examined using a phylogenetic generalised least-squares approach [following [65]]. Two PGLS analyses were undertaken in R software using the APE package [66] using either plant or insect phylogenetic information. Each model was implemented either with or without phylogenetic adjustment. Because most of the branch lengths in the phylogenies are unknown, the PGLS analysis was conducted with the assumption that all branches in the phylogeny were of equal length. This is equivalent to a punctuated model of evolution in which all change occurs at speciation events. The most appropriate model was selected by assessment of the Akaike Information Criterion (AIC) and Akaike weights (*w*_*i*_) according to the information theoretic approach outlined in Burnham and Anderson [67]. Accordingly, models with the lowest AIC values were assumed to be better than models with high AIC values. In order to standardize the directions of slope deviations we present all regressions (at both the population and species level) with plant traits on the X-axis. We chose to report our results only in this manner because lower AIC values for PGLS suggest that this is the most suitable model. These analyses were also repeated with RMA without correction for phylogenetic non-independence.

The hypothetical plant phylogeny used in the PGLS was built to the family level following [68]. Species-level phylogenetic information was obtained for the Iridaceae [69], Orchidaceae [70] and Geraniaceae [71] using the strict consensus tree in each case. In a few cases, taxa which could not be resolved from these sources were assigned hypothetical phylogenetic relationships assuming species within genera are most closely related [as in [72]]. The final plant phylogeny used in the PGLS is given (Additional file 3).

A hypothetical insect phylogeny for use in the PGLS was built following Terblanche et al. [72]. Taxa were resolved to the order level [73], to the species level for the Sphingidae [74] (Additional file 3) and to the family level for Hymenoptera and Hemiptera following the Tree of Life project http://www.tolweb.org/tree. Diptera families were resolved following the Tree of Life and Tabanidae species resolved according to Morita [75]. Nemestrinidae formed a large polytomy which could only be partially resolved assuming species within genera are more related than those among genera. The final insect phylogeny used in the PGLS is given in Additional file 3.

#### The effect of plant breeding system on trait matching

We tested this idea using the larger species-level data set which included 98 pollinator-dependent plants and 12 autonomous selfers; only pollination relationships were used for this analysis. Plants with unknown breeding systems were excluded from the analysis. We separately regressed tube lengths with pollinator morphology for all species which were dependent on pollinators for seed set, and then for autonomous selfers which were not dependent on pollinators for seed set. We then compared these regression slopes to one another using a homogeneity of slopes test in a generalized linear model (GLZ) with a normal distribution and log link function. Here plant tube length was placed on the Y-axis as we were using plant mating system as a categorical predictor of tube-length. The data did not satisfy the assumptions of non-independence of data and homogeneity of variance for analyses by GLM, which is why we used GLZ, without phylogenetic adjustment, which is more conservative and robust to these errors. We expected that plants reliant on pollinators for seed set are more likely to be ahead in the coevolutionary arms race (i.e. to match or exceed pollinator magnitudes) than autonomous selfers, which likely experience weaker selection to match pollinator magnitudes.

## Authors' contributions

BA conceived of the original idea, contributed to the writing and certain analyses. AGE participated in the writing of the manuscript, helped with the interpretation of the data and was the major source of critical thought. JST completed most of the statistical analyses, contributed to the writing and extended the original idea of the manuscript. All authors read and approved the final manuscript.

## Supplementary Material

Additional file 1 **Summary of ordinary least-squares (OLS) regression results for tests of morphological symmetry in adaptive traits within and among reported study systems**. In all cases the plant's morphological trait is the independent variable while the insect's morphological trait is the dependent variable. Note that the origin of the regression was allowed to vary freely (i.e. not constrained to zero). Plant breeding system is indicated for pollination relationships with O = outcrossing and AS = Autonomous selfing, and the type of interaction is categorized as parasitic or pollination. Pollination relationships are further divided into mutualistic (M) or antagonistic (A) relationships. Preg = probability value for the F-value test for regression significance, Pslope = probability value for the comparison of estimated slope against expected unity (a value of 1.0), DF = degrees of freedom. The slope for "all populations" was derived by pooling all data points within each of the studies below, and not by averaging the slopes of each relationship. Insect order is indicated by a letter in parentheses where C = Coleoptera, H = Hemiptera, D = Diptera, Hy = Hymenoptera. † Bold values are significant at p = 0.05 after False Discovery Rate correction. Click here for file

Additional file 2 **List of taxa and source references used for interspecific comparisons**. When data in the original source reference was presented as a range the mid-point was assumed representative of the species mean trait value. If an insect species was found to interact with one or more plant species, each interaction was assumed to be an independent relationship for the interspecific comparison. However, this potential pseudo-replication of species data was fully accounted for in the PGLS analyses since multiple reports of the same species was represented as a polytomy in the hypothetical phylogeny, which therefore collapses to the nearest node in the tree in the phylogenetically-adjusted comparison and does not artificially inflate the degrees of freedom. Insect order is indicated by a letter in parentheses where C = Coleoptera, H = Hemiptera, D = Diptera, Hy = Hymenoptera, L = Lepidoptera. Click here for file

Additional file 3 **Inter-specific plant and insect phylogenies. Hypothesized phylogenetic relationships (in Newick format) used in PGLS analyses**. Owing to limited available information, all branch lengths were assumed to equal 1. Click here for file

## References

[B1] Darwin CR On the origin of species by means of natural selection, or the preservation of favoured races in the struggle for life 1859 1 Cambridge: Harvard University Press PMC5184128 30164232

[B2] Darwin CR On the various contrivances by which British and foreign orchids are fertilized by insects 1862 London: John Murray PMC5180317 30163543

[B3] Nilsson LA The evolution of flowers with deep corolla tubes Nature 1988 334 147 149 10.1038/334147a0

[B4] Rothschild LW Jordan K A revision of the lepidopterous family Sphingidae Novitates Zoologicae 1903 9 1 972

[B5] Mayhew PJ Discovering Evolutionary Ecology: Bringing Together Ecology and Evolution 2006 Oxford: Oxford University Press

[B6] Anderson B Johnson SD The geographical mosaic of coevolution in a plant-pollinator mutualism Evolution 2008 62 220 225 18067570 10.1111/j.1558-5646.2007.00275.x

[B7] Steiner KE Whitehead VB Oil flowers and oil bees: further evidence for pollinator adaptation Evolution 1991 45 1493 1501 10.2307/2409895 28563824

[B8] Steiner KE Whitehead VB Pollinator adaptation to oil-secreting flowers--*Redivivia *and *Diascia* Evolution 1990 44 1701 1707 10.2307/2409348 28564320

[B9] Berenbaum MR Zangerl AR Chemical phenotype matching between a plant and its insect herbivore Proc Natl Acad Sci USA 1998 95 13743 13748 10.1073/pnas.95.23.13743 9811871 PMC24890

[B10] Toju H Sota T Imbalance of predator and prey armament: geographic clines in phenotypic interface and natural selection Am Nat 2006 167 105 117 10.1086/498277 16475103

[B11] Pauw A Stofberg J Waterman RJ Flies and Flowers in Darwin's Race Evolution 2009 63 268 279 10.1111/j.1558-5646.2008.00547.x 19146595

[B12] Anderson B Alexandersson R Johnson SD Evolution and coexistence of pollination ecotypes in an African *Gladiolus *(Iridaceae) Evolution 2010 64 960 972 10.1111/j.1558-5646.2009.00880.x 19891625

[B13] Brodie ED Jr Ridenhour BJ Brodie ED II The evolutionary response of predators to dangerous prey: hotspots and coldspots in the geographic mosaic of coevolution between garter snakes and newts Evolution 2002 56 2067 2082 12449493 10.1111/j.0014-3820.2002.tb00132.x

[B14] Toju H Sota T Adaptive divergence of scaling relationships mediates the arms race between a weevil and its host plant Biol Lett 2006 2 539 542 10.1098/rsbl.2006.0514 17148283 PMC1833982

[B15] Whittall JB Hodges SA Pollinator shifts drive increasingly long nectar spurs in columbine flowers Nature 2007 447 706 710 10.1038/nature05857 17554306

[B16] Anderson B Johnson SD Carbutt C Exploitation of a specialized mutualism by a deceptive orchid Am J Bot 2005 92 1342 1349 10.3732/ajb.92.8.1342 21646154

[B17] Anderson B Johnson SD Geographical covariation and local convergence of flower depth in a guild of fly-pollinated plants New Phytologist 2009 182 533 540 10.1111/j.1469-8137.2009.02764.x 19210717

[B18] Janzen DH When is it coevolution? Evolution 1980 34 611 612 10.2307/2408229 28568694

[B19] Ridley M Evolution 2003 3 Blackwell Publishing

[B20] Thompson JN The Coevolutionary Process 1994 Chicago: University of Chicago Press

[B21] Thompson JN The Geographic Mosaic of Coevolution 2005 Chicago: University of Chicago Press

[B22] Thompson JN Nuismer SL Gomulkiewicz R Coevolution and maladaptation Integrative and Comparative Biology 2002 42 381 387 10.1093/icb/42.2.381 21708731

[B23] Brodie ED Ridenhour BJ Reciprocal selection at the phenotypic interface of coevolution Integrative and Comparative Biology 2003 43 408 418 10.1093/icb/43.3.408 21680449

[B24] Abrams PA Adaptive responses of predators to prey and prey to predators - the failure of the arms-race analogy Evolution 1986 40 1229 1247 10.2307/2408950 28563514

[B25] Dawkins R Krebs JR Arms races between and within species Proc Roy Soc Ser B 1979 205 489 511 10.1098/rspb.1979.0081 42057

[B26] Brodie ED Predator-prey arms races Bioscience 1999 49 557 568 10.2307/1313476

[B27] Bascompte J Jordano P Melian CJ Olesen JM The nested assembly of plant-animal mutualistic networks Proc Natl Acad Sci USA 2003 100 9383 87 10.1073/pnas.1633576100 12881488 PMC170927

[B28] Vazquez DP Aizen MA Asymmetric specialization: a pervasive feature of plant-pollinator interactions Ecology 2004 85 1251 1257 10.1890/03-3112

[B29] Bascompte J Jordano P Plant-animal mutualistic networks: the architecture of biodiversity Annu Rev Ecol Evol Syst 2007 38 567 93 10.1146/annurev.ecolsys.38.091206.095818

[B30] Bascompte JP Jordano P Olesen JM Asymmetric coevolutionary networks facilitate biodiversity maintenance Science 2006 312 431 433 10.1126/science.1123412 16627742

[B31] Jordano P Patterns of mutualistic interactions in pollination and seed dispersal: connectance, dependence asymmetries, and coevolution Am Nat 1987 129 657 77 10.1086/284665

[B32] Pauw A Floral syndromes accurately predict pollination by a specialized oil-collecting bee (*Rediviva peringueyi*, Melittidae) in a guild of South African orchids (Coryciinae) Amer J Bot 2006 93 917 926 10.3732/ajb.93.6.917 21642155

[B33] Lively CM Dybdahl MF Parasite adaptation to locally common host genotypes Nature 2000 405 679 681 10.1038/35015069 10864323

[B34] Benkman CW Holimon WC Smith JW The influence of a competitor on the geographic mosaic of coevolution between crossbills and lodgepole pine Evolution 2001 55 282 294 11308086 10.1111/j.0014-3820.2001.tb01293.x

[B35] Thompson JN Cunningham BM Geographic structure and dynamics of coevolutionary selection Nature 2002 417 735 738 10.1038/nature00810 12066183

[B36] Zangerl AR Berenbaum MR Phenotype matching in wild parsnip and parsnip webworms: Causes and consequences Evolution 2003 57 806 815 12778550 10.1111/j.0014-3820.2003.tb00292.x

[B37] Siepielski AM Benkman CW A role for habitat area in the geographic mosaic of coevolution between red crossbills and lodgepole pines J Evol Biol 2005 18 1042 1049 10.1111/j.1420-9101.2005.00902.x 16033577

[B38] Decaestecker E Gaba S Raeymaekers JAM Stoks R Van Kerckhoven L Ebert D De Meester L Host-parasite 'Red Queen' dynamics archived in pond sediment Nature 2007 460 870 873 10.1038/nature06291 18004303

[B39] Hanifin CT Brodie ED Jr Brodie ED II Phenotypic mismatches reveal escape from arms-race coevolution PLoS Biol 2008 6 471 482 10.1371/journal.pbio.0060060 PMC2265764 18336073

[B40] Gomulkiewicz R Thompson JN Holt RD Nuismer SL Hochberg ME Hot spots, cold spots, and the geographic mosaic theory of coevolution Am Nat 2000 156 156 174 10.1086/303382 10856199

[B41] Kunte K Allometry and functional constraints on proboscis lengths in butterflies Funct Ecol 2007 21 982 987 10.1111/j.1365-2435.2007.01299.x

[B42] Harder LD Flower handling efficiency of bumble bees: morphological aspects of probing time Oecologia 1983 57 274 280 10.1007/BF00379591 28310186

[B43] Borrell BJ Scaling of nectar foraging in orchid bees Am Nat 2007 169 569 580 10.1086/512689 17427129

[B44] Harder LD Morphology as a predictor of flower choice by bumble bees Ecology 1985 66 198 210 10.2307/1941320

[B45] Corbet SA Butterfly nectaring flowers: butterfly morphology and flower form Entomologia Experimentalis Et Applicata 2000 96 289 298 10.1023/A:1004096432758

[B46] Haber WA Frankie GW A tropical hawkmoth community - Costa Rican dry forest Sphingidae Biotropica 1989 21 155 172 10.2307/2388706

[B47] Minckley RL Cane JH Kervin L Roulston TH Spatial predictability and resource specialization of bees (Hymenoptera: Apoidea) at a superabundant, widespread resource Biol J Linn Soc 1999 67 119 147 10.1111/j.1095-8312.1999.tb01933.x

[B48] Thompson JN Mutualistic webs of species Science 2006 312 372 73 10.1126/science.1126904 16627726

[B49] Rezende EL Jordano P Bascompte J Effects of phenotypic complementarity and phylogeny on the nested structure of mutualistic networks Oikos 2007 116 1919 1929 10.1111/j.0030-1299.2007.16029.x

[B50] Santamarıa L Rodrıguez-Girones M Linkage rules for plant-pollinator networks: Trait complementarity or exploitation barriers? PLoS Biol 2007 5 e31 10.1371/journal.pbio.0050031 17253905 PMC1779813

[B51] Emlen DJ Nijhout HF The development and evolution of exaggerated morphologies in insects Ann Rev Entomol 2000 45 661 708 10.1146/annurev.ento.45.1.661 10761593

[B52] Nijhout HF The control of body size in insects Dev Biol 2003 261 1 9 10.1016/S0012-1606(03)00276-8 12941617

[B53] Terblanche JS Klok CJ Marais E Chown SL Metabolic rate in the whip-spider, *Damon annulatipes *(Arachnida: Amblypygi) J Insect Physiol 2004 50 637 645 10.1016/j.jinsphys.2004.04.010 15234624

[B54] Niven JE Scharlemann JPW Do insect metabolic rates at rest and during flight scale with body mass? Biol Lett 2005 1 346 349 10.1098/rsbl.2005.0311 17148203 PMC1617160

[B55] Chown SL Marais E Terblanche JS Klok CJ Lighton JRB Blackburn TM Scaling of insect metabolic rate is inconsistent with the nutrient supply network model Funct Ecol 2007 21 282 290 10.1111/j.1365-2435.2007.01245.x

[B56] Rezende EL Lavabre JE Guimaraes PR Jordano P Bascompte L Non-random coextinctions in phylogenetically structured mutualistic networks Nature 2007 448 925 929 10.1038/nature05956 17713534

[B57] Morjan CL Rieseberg LH How species evolve collectively: implications of gene flow and selection for the spread of advantageous alleles Mol Ecol 2004 13 1341 1356 10.1111/j.1365-294X.2004.02164.x 15140081 PMC2600545

[B58] Carroll SP Boyd C Host race radiation in the soapberry bug--natural history with the history Evolution 1992 46 1052 1069 10.2307/2409756 28564420

[B59] Bond WJ Do mutualisms matter? Assessing the impact of pollinator and disperser disruption on plant extinction Phil Trans Roy Soc Lond B 1994 344 83 90 10.1098/rstb.1994.0055

[B60] Hegland SJ Nielsen A Lazaro A Bjerknes AL Totland O How does climate warming affect plant-pollinator interactions? Ecol Lett 2009 12 184 195 10.1111/j.1461-0248.2008.01269.x 19049509

[B61] Warton DI Wright IJ Falster DS Westoby M Bivariate line-fitting methods for allometry Biol Rev 2006 81 259 291 10.1017/S1464793106007007 16573844

[B62] Zar JH Biostatistical Analysis 1999 4 New Jersey: Prentice Hall

[B63] Bohonak AJ van der Linde K RMA: Software for Reduced Major Axis regression Java version 2004 http://www.kimvdlinde.com/professional/rma.html

[B64] Benjamini Y Hochberg Y Controlling the false discovery rate: a practical and powerful approach to multiple testing J Royal Stat Soc B 1995 57 289 300

[B65] Halsey LG Butler PJ Blackburn TM A phylogenetic analysis of the allometry of diving Am Nat 2006 167 276 287 10.1086/499439 16670986

[B66] Paradis E Claude J Strimmer K APE: analysis of phylogenetics and evolution in R language Bioinformatics 2004 20 289 290 10.1093/bioinformatics/btg412 14734327

[B67] Burnham KP Anderson DR Kullback-Leibler information as a basis for strong inference in ecological studies Wildlife Research 2001 28 111 119 10.1071/WR99107

[B68] Davies TJ Barraclough TG Chase MW Soltis PS Soltis DE Savolainen V Darwin's abominable mystery: Insights from a supertree of the angiosperms Proc Natl Acad Sci USA 2004 101 1904 1909 10.1073/pnas.0308127100 14766971 PMC357025

[B69] Goldblatt P Savolainen V Porteous O Sostaric I Powell M Reeves G Manning JC Barraclough TG Chase MW Radiation in the Cape flora and the phylogeny of peacock irises Moraea (Iridaceae) based on four plastid DNA regions Mol Phyl Evol 2002 25 341 360 10.1016/S1055-7903(02)00235-X 12414315

[B70] Bytebier B Bellstedt DU Linder HP A molecular phylogeny for the large African orchid genus *Disa* Mol Phyl Evol 2007 43 75 90 10.1016/j.ympev.2006.08.014 17081772

[B71] Bakker FT Culham A Hettiarachi P Touloumenidou T Gibby M Phylogeny of *Pelargonium *(Geraniaceae) based on DNA sequences from three genomes Taxon 2004 53 17 28 10.2307/4135485

[B72] Terblanche JS White CR Blackburn TM Marais E Chown SL Scaling of gas exchange cycle frequency in insects Biol Lett 2008 4 127 129 10.1098/rsbl.2007.0522 18055409 PMC2412941

[B73] Gullan PJ Cranston PC The Insects. An Outline of Entomology 2005 UK: Blackwell Publishing

[B74] Kitching IJ The phylogenetic relationships of Morgan's Sphinx, *Xanthopan morganii *(Walker), the tribe Acherontiini, and allied long-tongued hawkmoths (Lepidoptera: Sphingidae, Sphinginae) Zool J Linn Soc 2002 135 471 527 10.1046/j.1096-3642.2002.00021.x

[B75] Morita SI A phylogeny of long-tongued horse flies (*Philolice*, Diptera: Tabanidae) with the first cladistic review of higher relationships within the family Invert Syst 2008 22 311 327 10.1071/IS07005

[B76] Johnson SD Steiner KE Long-tongued fly pollination and evolution of floral spur length in the *Disa draconis *complex (Orchidaceae) Evolution 1997 51 45 53 10.2307/2410959 28568792

[B77] Goldblatt P Bernhardt P Pollination biology of Nivenia (Iridaceae) and the presence of heterostylous self compatibility Isr J Bot 1990 39 93 111

[B78] Goldblatt P Manning JC The long-proboscid fly pollination system in *Gladiolus *(Iridaceae) Ann Miss Bot Gard 1999 86 758 774 10.2307/2666153

[B79] Manning JC Goldblatt P The *Moegistorhynchus longirostris *(Diptera: Nemistrinidae) pollination guild: long-tubed flowers and a specialized long-proboscid fly pollination system in southern Africa Plant Syst Evol 1997 206 51 69 10.1007/BF00987941

[B80] Goldblatt P Manning JC Bernhardt P Pollination biology of *Lapeirousia *subgenus *Lapeirousia *(Iridaceae) in southern Africa: Floral divergence and adaption for long-tongued fly pollination Ann Miss Bot Gard 1995 82 517 534 10.2307/2399833

[B81] Manning JC Goldblatt P The *Prosoeca peringueyi *(Diptera: Nemistrinidae) pollination guild in southern Africa: Long tongued flies and their tubular flowers Ann Miss Bot Gard 1996 83 67 86 10.2307/2399969

[B82] Goldblatt P Manning JC The long-proboscid fly pollination system in southern Africa Ann Miss Bot Gard 2000 87 146 170 10.2307/2666158

[B83] Goldblatt P Bernhardt P Manning JC Adaptive radiation of pollination mechanisms in Ixia (Iridaceae: Crocoideae) Ann Miss Bot Gard 2000 87 564 577 10.2307/2666146

[B84] Grant V Grant KA Behavious of hawkmoths on flowers of *Datura meteloides* Bot Gazette 1983 144 280 284 10.1086/337374

[B85] Grant V Grant KA Hawkmoth pollination of *Mirabilis longiflora *(Nyctaginaceae) Proc Nat Acad Sci USA 1983 80 1298 1299 10.1073/pnas.80.5.1298 16593287 PMC393583

[B86] Gregory DP Hawkmoth pollination in the genus *Oenothera* Aliso 1963 5 4 357 419

[B87] Johnson SD Liltved WR Hawkmoth pollination of *Bonatea speciosa *(Orchidaceae) in a South African coastal forest Nord J Bot 1997 17 5 10 10.1111/j.1756-1051.1997.tb00286.x

[B88] Johnson SD Morita S Lying to Pinocchio: floral deception in an orchid pollinated by long-proboscid flies Bot J Linn Soc 2006 152 271 278 10.1111/j.1095-8339.2006.00571.x

[B89] Johnson SD Steiner KE Long-proboscid fly pollination of 2 orchids in the Cape-Drakensberg mountains, South-Africa Pl Syst Evol 1995 195 169 175 10.1007/BF00989293

[B90] Johnson SD Evidence for Batesian mimicry in a butterfly-pollinated orchid Biol J Linn Soc 1994 53 91 104

[B91] Johnson SD Observations of hawkmoth pollination in the South-African orchid *Disa-cooperi* Nord J Bot 1995 15 121 125 10.1111/j.1756-1051.1995.tb00128.x

[B92] Johnson SD Batesian mimicry in the non-rewarding orchid *Disa pulchra*, and its consequences for pollinator behaviour Biol J Linn Soc 2000 71 119 132 10.1006/bijl.1999.0430

[B93] Johnson SD Pollination by long-proboscid flies in the endangered African orchid *Disa skullyi* S Afr J Bot 2006 72 24 27 10.1016/j.sajb.2005.04.002

[B94] Johnson SD Edwards TJ Carbutt C Potgieter C Specialization for hawkmoth and long-proboscid fly pollination in *Zalusianskya *section Nycterinia (Scrophulariaceae) Bot J Linn Soc 2002 138 17 27 10.1046/j.1095-8339.2002.00005.x

[B95] Johnson SD Alexandersson R Linder HP Experimental and phylogenetic evidence for floral mimicry in a guild of fly-pollinated plants Biol J Linn Soc 2003 80 289 304 10.1046/j.1095-8312.2003.00236.x

[B96] Manning JC Goldblatt P Cupid comes in many guises: The not-so-humble fly and a pollination guild in the Overberg Veld & Flora 1995 81 50 53

[B97] Struck M Floral divergence and convergence in the genus *Pelargonium *(Geraniaceae) in southern Africa: ecological and evolutionary considerations Pl Syst Evol 1997 208 71 97 10.1007/BF00986083

[B98] Miller RB Hawkmoths and the geographic patterns of floral variation in *Aquilegia caerulea* Evolution 1981 35 763 774 10.2307/2408246 28563131

[B99] Nilsson LA Rabakonandrianina E Hawk-moth scale analysis and pollination specialization in the epilithic malagasy endemic *Aerangis-ellisii *(Reichenb fil) Schltr (Orchidaceae) Bot J Linn Soc 1988 97 49 61 10.1111/j.1095-8339.1988.tb01686.x

[B100] Nilsson LA Jonsson L Ralison L Randrianjohany E Angrecoid orchids and hawkmoths in central Madagascar - Specialized pollination systems and generalist foragers Biotropica 1987 19 310 318 10.2307/2388628

[B101] Potgieter CJ Edwards TJ Miller RM van Staden J Pollination of seven *Plectranthus *spp. (Lamiaceae) in southern Natal, South Africa Pl Syst Evol 1999 218 99 112 10.1007/BF01087038

