# Supplementary material for: Predictable patterns of trait mismatches between interacting plants and insects
Source: BMC Evol Biol. 2010 Jul 7;10:204. doi: 10.1186/1471-2148-10-204 (PMC2927919; doi:10.1186/1471-2148-10-204)
Supplement: Additional file 1 — Summary of ordinary least-squares (OLS) regression results for tests of morphological symmetry in adaptive traits within and among reported study systems. In all cases the plant's morphological trait is the independent variable while the insect's morphological trait is the dependent variable. Note that the origin of the regression was allowed to vary freely (i.e. not constrained to zero). Plant breeding system is indicated for pollination relationships with O = outcrossing and AS = Autonomous selfing, and the type of interaction is categorized as parasitic or pollination. Pollination relationships are further divided into mutualistic (M) or antagonistic (A) relationships. Preg = probability value for the F-value test for regression significance, Pslope = probability value for the comparison of estimated slope against expected unity (a value of 1.0), DF = degrees of freedom. The slope for "all populations" was derived by pooling all data points within each of the studies below, and not by averaging the slopes of each relationship. Insect order is indicated by a letter in parentheses where C = Coleoptera, H = Hemiptera, D = Diptera, Hy = Hymenoptera. † Bold values are significant at p = 0.05 after False Discovery Rate correction. [file 1471-2148-10-204-S1.DOC]

| Insect | Plant | Reference | Co-evolved/  Unilateral | Breeding system | Interaction | Slope ± SE | Intercept ± SE | R^2^ | Preg | t | Pslope^†^ | DF |
| --- | --- | --- | --- | --- | --- | --- | --- | --- | --- | --- | --- | --- |
| *Curculio camelliae* (C) | *Camellia japonica* | [10] | C | NA | Parasitic | 0.6623 ± 0.137 | 5.4601 ± 1.0249 | 0.744 | 0.0013 | 2.461 | **0.01308** | 13 |
| *Jadera haematoloma* (H) | Sapindaceae | [58] | ? | NA | Parasitic | 0.2456 ± 0.0503 | 5.9002 ± 0.3556 | 0.857 | 0.0081 | 14.998 | **0.000058** | 4 |
| *Tabanid* sp. (D) | *Disa draconis* | [76] | U | O | Pollination (A) | 0.4540 ± 0.0875 | 9.4300 ± 3.0421 | 0.951 | 0.0247 | 5.189 | **0.006950** | 2 |
| *Rediviva neliana* (Hy) | *Diascia* spp. | [7] | C | O | Pollination (M) | 0.6015 ± 0.0441 | 6.3480 ± 0.4305 | 0.907 | 0.0001 | 9.036 | **0.000001** | 19 |
| *Rediviva neliana* (Hy) | *Diascia capsularis* | [7] | C | O | Pollination (M) | 0.7191 ± 0.2002 | 4.8555 ± 2.4315 | 0.721 | 0.0157 | 1.403 | 0.109780 | 5 |
| *Rediviva pallidula* (Hy) | *Diascia* spp. | [7] | C | O | Pollination (M) | 0.4406 ± 0.0650 | 8.0694 ± 0.6180 | 0.920 | 0.0025 | 8.606 | **0.000175** | 4 |
| *Prosoeca ganglbaueri* (D) | *Disa nivea* | [16] | U | O | Pollination (A) | 0.5882 ± 0.1931 | 17.4893 ± 7.8299 | 0.699 | 0.0382 | 2.133 | 0.051072 | 4 |
| *P. ganglbaueri* (D) | *Zalusianskya microsiphon* | [6] | C | O | Pollination (M) | 0.6531 ± 0.1172 | 11.3384 ± 4.4998 | 0.689 | 0.0001 | 2.960 | **0.005170** | 14 |
| *P. ganglbaueri* (D) | *Gladiolus oppositiflorus* | [17] | ? | O | Pollination (M) | 0.6885 ± 0.1628 | 14.3605 ± 6.9528 | 0.817 | 0.0134 | 1.913 | 0.064151 | 4 |
| *P. ganglbaueri* (D) | *Amaryllis* spp. | [17] | ? | O | Pollination (M) | 0.8143 ± 0.1892 | 3.2058 ± 7.7566 | 0.861 | 0.0231 | 0.982 | 0.199256 | 3 |
| *Moegisterynchus longirostris* (D) | *Lapeirousia anceps* | [32] | C | AS | Pollination (M) | 1.0004 ± 0.3247 | 8.6527 ± 18.9796 | 0.613 | 0.0216 | -0.001 | 0.999 | 6 |
| *M. longirostris* (D) | *Babiana tubulosa* | [32] | ? | AS | Pollination (M) | 1.0980 ± 0.5176 | -18.1060 ± 40.5308 | 0.600 | 0.1240 | -0.189 | 0.862 | 3 |
