# Supplementary material for: Predictable patterns of trait mismatches between interacting plants and insects
Source: BMC Evol Biol. 2010 Jul 7;10:204. doi: 10.1186/1471-2148-10-204 (PMC2927919; doi:10.1186/1471-2148-10-204)
Supplement: Additional file 2 — List of taxa and source references used for interspecific comparisons. When data in the original source reference was presented as a range the mid-point was assumed representative of the species mean trait value. If an insect species was found to interact with one or more plant species, each interaction was assumed to be an independent relationship for the interspecific comparison. However, this potential pseudo-replication of species data was fully accounted for in the PGLS analyses since multiple reports of the same species was represented as a polytomy in the hypothetical phylogeny, which therefore collapses to the nearest node in the tree in the phylogenetically-adjusted comparison and does not artificially inflate the degrees of freedom. Insect order is indicated by a letter in parentheses where C = Coleoptera, H = Hemiptera, D = Diptera, Hy = Hymenoptera, L = Lepidoptera. [file 1471-2148-10-204-S2.DOC]

| **Insect species** | **Trait** | **Plant species** | **Trait** | **Reference** |
| --- | --- | --- | --- | --- |
| *Prosoeca ganglbaueri* (D) | Proboscis length | *Zaluzianskya microsiphon* | Corolla length | [6] |
| *Jadera haematoloma* (H) | Beak length | *Sapindaceae spp.* | Fruit radius | [58] |
| *Xanthopan morgani praedicta* (L) | Proboscis length | *Angraecum sesquipedale* | Spur length | [2, 15] |
| *Prosoeca ganglbaueri* (D) | Proboscis length | *Nivenia stenosiphon* | Perianth length | [77] |
| *Moegistorynchus* sp. nov. (D) | Proboscis length | *Gladiolus rhodanthus* | Perianth length | [78] |
| *Philoliche gulosa* (D) | Proboscis length | *Gladiolus floribundus* | Perianth length | [78] |
| *Philoliche rostrata* (D) | Proboscis length | *Gladiolus carneus* | Perianth length | [78] |
| *Philoliche rostrata* (D) | Proboscis length | *Gladiolus floribundus* | Perianth length | [78] |
| *Philoliche rostrata* (D) | Proboscis length | *Gladiolus monticola* | Perianth length | [78] |
| *Philoliche rostrata* (D) | Proboscis length | *Gladiolus vigilans* | Perianth length | [78] |
| *Philoliche rostrata* (D) | Proboscis length | *Gladiolus virgatus* | Perianth length | [78] |
| *Proscoeca nitidula* (D) | Proboscis length | *Gladiolus carneus* | Perianth length | [78] |
| *Proscoeca nitidula* (D) | Proboscis length | *Gladiolus monticola* | Perianth length | [78] |
| *Prosoeca ganglbaueri* (D) | Proboscis length | *Gladiolus microcarpus* | Perianth length | [78] |
| *Prosoeca ganglbaueri* (D) | Proboscis length | *Gladiolus mortonius* | Perianth length | [78] |
| *Prosoeca ganglbaueri* (D) | Proboscis length | *Gladiolus varius* | Perianth length | [78] |
| *Prosoeca longipennis* (D) | Proboscis length | *Gladiolus engysiphon* | Perianth length | [78, 79] |
| *Prosoeca robusta* (D) | Proboscis length | *Gladiolus calcaratus* | Perianth length | [80, 81] |
| *Stenobasipteron wiedemannii* (D) | Proboscis length | *Gladiolus macneilii* | Perianth length | [80, 81] |
| *Philoliche rostrata* (D) | Proboscis length | *Gladiolus undulatus* | Perianth length | [80, 81] |
| *Proscoeca* sp. nov*.* (D) | Proboscis length | *Babiana flabellifolia* | Perianth length | [80, 81] |
| *Proscoeca* sp. nov. (D) | Proboscis length | *Babiana framesii* | Perianth length | [80, 81] |
| *Proscoeca* sp. nov*.* (D) | Proboscis length | *Lapeirousia jacquinii* | Perianth length | [80, 81] |
| *Proscoeca* sp. nov. (D) | Proboscis length | *Lapeirousia oreogena* | Perianth length | [80, 81] |
| *Proscoeca* sp. nov. (D) | Proboscis length | *Romulea hantamensis* | Perianth length | [80, 81] |
| *Proscoeca peringueyi* (D) | Proboscis length | *Babiana dredgei* | Perianth length | [80, 81] |
| *Proscoeca peringueyi* (D) | Proboscis length | *Babiana framesii* | Perianth length | [80, 81] |
| *Proscoeca peringueyi* (D) | Proboscis length | *Babiana geniculata* | Perianth length | [80, 81] |
| *Proscoeca peringueyi* (D) | Proboscis length | *Babiana pubescens* | Perianth length | [80, 81] |
| *Proscoeca peringueyi* (D) | Proboscis length | *Hesperantha latifolia* | Perianth length | [80, 81] |
| *Proscoeca peringueyi* (D) | Proboscis length | *Lapeirousia dolomitica* | Perianth length | [80, 81] |
| *Proscoeca peringueyi* (D) | Proboscis length | *Lapeirousia jacquinii* | Perianth length | [80, 81] |
| *Proscoeca peringueyi* (D) | Proboscis length | *Lapeirousia pyramidalis regalus* | Perianth length | [80, 81] |
| *Proscoeca peringueyi* (D) | Proboscis length | *Lapeirousia silenoiedes* | Perianth length | [80, 81] |
| *Proscoeca peringueyi* (D) | Proboscis length | *Lapeirousia violacea* | Perianth length | [80, 81] |
| *Proscoeca peringueyi* (D) | Proboscis length | *Pelargonium incrussatum* | Hypanthium length | [80, 81] |
| *Proscoeca peringueyi* (D) | Proboscis length | *Pelargonium magenteum* | Hypanthium length | [80, 81] |
| *Proscoeca peringueyi* (D) | Proboscis length | *Pelargonium sericifolium* | Hypanthium length | [80, 81] |
| *Proscoeca peringueyi* (D) | Proboscis length | *Sparaxis metelerkampiae* | Perianth length | [80, 81] |
| *Proscoeca peringueyi* (D) | Proboscis length | *Babiana curviscapa* | Perianth length | [80, 81, 82] |
| *Philoliche gulosa* (D) | Proboscis length | *Pelargonium elongatum* | Hypanthium length | [80, 81] |
| *Moegistorynchus longirostris* (D) | Proboscis length | *Lapeirousia anceps* | Perianth length | [79, 80, 81] |
| *Moegistorynchus longirostris* (D) | Proboscis length | *Lapeirousia fabricii* | Perianth length | [79, 80, 81] |
| *Philoliche gulosa* (D) | Proboscis length | *Geissorhiza confusa* | Perianth length | [82] |
| *Philoliche gulosa* (D) | Proboscis length | *Lobelia coronopifolia* | Corolla length | 10 |
| *Philoliche gulosa* (D) | Proboscis length | *Pelargonium oxyphyllum* | Hypanthium length | [82] |
| *Philoliche gulosa* (D) | Proboscis length | *Pelargonium peltatum* | Hypanthium length | [82] |
| *Philoliche gulosa* (D) | Proboscis length | *Tritonia flabellifolia* | Perianth length | [82] |
| *Philoliche rostrata* (D) | Proboscis length | *Geissorhiza bonaspei* | Perianth length | [82] |
| *Philoliche rostrata* (D) | Proboscis length | *Geissorhiza confusa* | Perianth length | [82] |
| *Philoliche rostrata* (D) | Proboscis length | *Pelargonium elongatum* | Hypanthium length | [82] |
| *Philoliche rostrata* (D) | Proboscis length | *Tritonia flabellifolia* | Perianth length | [82] |
| *Philoliche rostrata* (D) | Proboscis length | *Tritonia pallida* | Perianth length | [82] |
| *Proscoeca nitidula* (D) | Proboscis length | *Geissorhiza bonaspei* | Perianth length | [82] |
| *Proscoeca nov.* (D) | Proboscis length | *Babiana sambucina var longibracteata* | Perianth length | [82] |
| *Proscoeca peringueyi* (D) | Proboscis length | *Babiana ecklonii* | Perianth length | [82] |
| *Proscoeca peringueyi* (D) | Proboscis length | *Babiana sambucina var unguiculata* | Perianth length | [82] |
| *Prosoeca ganglbaueri* (D) | Proboscis length | *Pelargonium carneum* | Hypanthium length | [82] |
| *Moegistorynchus* sp. nov. (D) | Proboscis length | *Watsonia paucifolia* | Perianth length | [82] |
| *Philoliche gulosa* (D) | Proboscis length | *Aristea spiralis* | Perianth length | [82] |
| *Philoliche gulosa* (D) | Proboscis length | *Ixia bellendenii* | Perianth length | [83] |
| *Philoliche gulosa* (D) | Proboscis length | *Ixia paucifolia* | Perianth length | [83] |
| *Philoliche rostrata* (D) | Proboscis length | *Ixia paucifolia* | Perianth length | [83] |
| *Prosoeca ganglbaueri* (D) | Proboscis length | *Disa amoena* | Spur length | [82] |
| *Prosoeca ganglbaueri* (D) | Proboscis length | *Gladiolus oppositiflorus* | Perianth length | [82] |
| *Prosoeca ganglbaueri* (D) | Proboscis length | *Hesperantha grandiflora* | Perianth length | [82] |
| *Prosoeca ganglbaueri* (D) | Proboscis length | *Hesperantha scopulosa* | Perianth length | [82] |
| *Prosoeca ganglbaueri* (D) | Proboscis length | *Hesperantha woodii* | Perianth length | [82] |
| *Prosoeca ganglbaueri* (D) | Proboscis length | *Pelargonium gracillimum* | Hypanthium length | [82] |
| *Prosoeca ganglbaueri* (D) | Proboscis length | *Tritoniopsis revoluta* | Perianth length | [82] |
| *Prosoeca ganglbaueri* (D) | Proboscis length | *Watsonia wilmsii* | Perianth length | [82] |
| *Prosoeca robusta* (D) | Proboscis length | *Disa amoena* | Spur length | [82] |
| *Prosoeca robusta* (D) | Proboscis length | *Gladiolus varius* | Perianth length | [82] |
| *Prosoeca robusta* (D) | Proboscis length | *Watsonia wilmsii* | Perianth length | [82] |
| *Stenobasipteron wiedemannii* (D) | Proboscis length | *Brownleea coerulea* | Spur length | [82] |
| *Stenobasipteron wiedemannii* (D) | Proboscis length | *Hesperantha brevicaulis* | Perianth length | [82] |
| *Stenobasipteron wiedemannii* (D) | Proboscis length | *Orthosiphon tubiformis* | Corolla length | [82] |
| *Manduca quinquemaculata* (L) | Proboscis length | *Datura meteloides* | Corolla length | [84] |
| *Manduca quinquemaculata* (L) | Proboscis length | *Mirabilis longiflora* | Perianth length | [85] |
| *Manduca quinquemaculata* (L) | Proboscis length | *Acleisanthes longiflora* | Corolla length | [86] |
| *Manduca quinquemaculata* (L) | Proboscis length | *Oenothera brachycarpa* | Hypanthium length | [86] |
| *Manduca quinquemaculata* (L) | Proboscis length | *Oenothera taraxacoides* | Hypanthium length | [86] |
| *Manduca rustica* (L) | Proboscis length | *Acleisanthes longiflora* | Corolla length | [86] |
| *Thereta capensis* (L) | Proboscis length | *Bonatea speciosa* | Spur length | [87] |
| *Philoliche aethiopica* (D) | Proboscis length | *Disa nervosa* | Spur length | [88] |
| *Philoliche aethiopica* (D) | Proboscis length | *Watsonia densifloria* | Perianth length | [88] |
| *Prosoeca ganglbaueri* (D) | Proboscis length | *Brownleea macroceras* | Spur length | [89] |
| *Prosoeca ganglbaueri* (D) | Proboscis length | *Disa oreophila* | Spur length | [89] |
| *Philoliche rostrata* (D) | Proboscis length | *Pelargonium longicaule* | Hypanthium length | [76] |
| *Philoliche rostrata* (D) | Proboscis length | *Pelargonium myrrhifolium* | Hypanthium length | [76] |
| *Moegistorynchus longirostris* (D) | Proboscis length | *Disa draconis* | Spur length | [76] |
| *Tabanid sp.* (D) | Proboscis length | *Disa harveiana* | Spur length | [76] |
| *Meneris tulbaghia* (L) | Proboscis length | *Disa ferruginea* | Spur length | [90] |
| *Basiotha schenki* (L) | Proboscis length | *Disa cooperi* | Spur length | [91] |
| *Philoliche aethiopica* (D) | Proboscis length | *Disa pulchra* | Spur length | [92] |
| *Philoliche aethiopica* (D) | Proboscis length | *Watsonia lepida* | Perianth length | [92] |
| *Prosoeca ganglbaueri* (D) | Proboscis length | *Disa skullyi* | Spur length | [93] |
| *Hippotion celerio* (L) | Proboscis length | *Zaluzianskya elongata* | Corolla length | [94] |
| *Hippotion celerio* (L) | Proboscis length | *Zaluzianskya natalensis* | Corolla length | [94] |
| *Hippotion celerio* (L) | Proboscis length | *Zaluzianskya pulvinata* | Corolla length | [94] |
| *Prosoeca* sp 2 (D) | Proboscis length | *Zaluzianskya microsiphon* | Corolla length | [94] |
| *Prosoeca* sp. 1 (D) | Proboscis length | *Disa cephalotes* | Spur length | [95] |
| *Prosoeca* sp.  *1* (D) | Proboscis length | *Scabiosa columbaria* | Corolla length | [95] |
| *Prosoeca longipennis* (D) | Proboscis length | *Gladiolus bilineatus* | Perianth length | [96] |
| *Prosoeca longipennis* (D) | Proboscis length | *Pelargonium carneum* | Hypanthium length | [96] |
| *Prosoeca longipennis* (D) | Proboscis length | *Pelargonium dipetalum* | Hypanthium length | [96] |
| *Prosoeca longipennis* (D) | Proboscis length | *Pelargonium pinnatum* | Hypanthium length | [96] |
| *Moegistorynchus longirostris* (D) | Proboscis length | *Babiana tubulosa* | Perianth length | [79] |
| *Moegistorynchus longirostris* (D) | Proboscis length | *Geissorhiza exscapa* | Perianth length | [79] |
| *Moegistorynchus longirostris* (D) | Proboscis length | *Gladiolus angustus* | Perianth length | [79] |
| *Moegistorynchus longirostris (*D) | Proboscis length | *Pelargonium longcaule* | Hypanthium length | [79] |
| *Moegistorynchus longirostris* (D) | Proboscis length | *Pelargonium praemorsum* | Hypanthium length | [79] |
| *Moegistorynchus longirostris* (D) | Proboscis length | *Tritonia crispa* | Perianth length | [79] |
| *Philoliche gulosa* (D) | Proboscis length | *Tritonia crispa* | Perianth length | [79] |
| *Moegistorynchus longirostris* (D) | Proboscis length | *Ixia panniculata* | Perianth length | [76, 79] |
| *Philoliche rostrata* (D) | Proboscis length | *Pelargonium patulum* | Hypanthium length | [79, 97] |
| *Hyles lineata* (L) | Proboscis length | *Aquilegia caerulea v. carulea* | Spur length | [98] |
| *Hyles lineata* (L) | Proboscis length | *Aquilegia caerulea v. pinetorum* | Spur length | [98] |
| *Sphinx vashti* (L) | Proboscis length | *Aquilegia caerulea v. pinetorum* | Spur length | [98] |
| *Agrius convolvuli* (L) | Proboscis length | *Aerangis ellisii* | Spur length | [99] |
| *Panogena lingens* (L) | Proboscis length | *Aerangis ellisii* | Spur length | [99] |
| *Panogena lingens* (L) | Proboscis length | *Angraecum arachnites* | Spur length | [100] |
| *Panogena lingens* (L) | Proboscis length | *Angraecum articulata* | Spur length | [100] |
| *Panogena lingens* (L) | Proboscis length | *Angraecum compactum* | Spur length | [100] |
| *Panogena lingens* (L) | Proboscis length | *Angraecum fuscata* | Spur length | [100] |
| *Panogena lingens* (L) | Proboscis length | *Jumellea teretifiola* | Spur length | [100] |
| *Panogena lingens* (L) | Proboscis length | *Neobathiea grandidierana* | Corolla length | [100] |
| *Stenobasipteron sp* (D) | Proboscis length | *Plectranthus ambiguus* | Corolla length | [101] |
| *Stenobasipteron sp* (D) | Proboscis length | *Plectranthus hilliardii* | Corolla length | [101] |
| *Stenobasipteron wiedemannii* (D) | Proboscis length | *Plectranthus ecklonii* | Corolla length | [78, 101] |
| *Rediviva neliana* (Hy) | Foreleg length | *Diascia capsularis* | Spur length | [7] |
| *Rediviva neliana* (Hy) | Foreleg length | *Diascia sp1* | Spur length | [7] |
| *Rediviva pallidula* (Hy) | Foreleg length | *Diascia sp2* | Spur length | [8] |
| *Philoliche rostrata* (D) | Proboscis length | *Pelargonium peltatum* | Hypanthium length | [79, 97] |
| *Curculio camelliae* (C) | Rostrum length | *Camellia japonica* | Pericarp thickness | [10] |
