# Supplementary material for: Predictable patterns of trait mismatches between interacting plants and insects
Source: BMC Evol Biol. 2010 Jul 7;10:204. doi: 10.1186/1471-2148-10-204 (PMC2927919; doi:10.1186/1471-2148-10-204)
Supplement: Additional file 3 — Inter-specific plant and insect phylogenies. Hypothesized phylogenetic relationships (in Newick format) used in PGLS analyses. Owing to limited available information, all branch lengths were assumed to equal 1. [file 1471-2148-10-204-S3.DOC]

**Inter-specific plant phylogeny**

(Sapindaceae_spp.:1,(Oenothera_brachycarpa:1,Oenothera_taraxacoides:1):1,((((Pelargonium_praemorsum:1,((Pelargonium_longicaule1:1,Pelargonium_longicaule2:1):1,Pelargonium_myrrhifolium:1):1):1,((Pelargonium_elongatum1:1,Pelargonium_elongatum2:1):1,(Pelargonium_peltatum1:1,Pelargonium_peltatum2:1):1):1):1,(Pelargonium_patulum:1,((Pelargonium_magenteum:1,Pelargonium_rotundidipetalum:1):1,(Pelargonium_sericifolium:1,(Pelargonium_a._carneum1:1,Pelargonium_a._carneum2:1):1):1):1):1,Pelargonium_gracillimum:1,Pelargonium_incrussatum:1,Pelargonium_oxyphyllum:1,Pelargonium_pinnatum:1):1,((Datura_meteloides:1,((((((Orthosiphon_tubiformis:1,(Plectranthus_ambiguus:1,Plectranthus_ecklonii:1,Plectranthus_hilliardii:1):1):1,(Lobelia_coronopifolia:1,Scabiosa_columbaria:1):1):1,Camellia_japonica:1):1,((Acleisanthes_longiflora1:1,Acleisanthes_longiflora2:1):1,Mirabilis_longiflora:1):1):1,(Aquilegia_caerulea_v._carulea:1,(Aquilegia_caerulea_v._pinetorum1:1,Aquilegia_caerulea_v._pinetorum2:1):1):1):1,((Diascia_capsularis:1,(Diascia_sp1:1,Diascia_sp2:1):1):1,((Zaluzianskya_microsiphon1:1,Zaluzianskya_microsiphon2:1,Zaluzianskya_microsiphon3:1):1,Zaluzianskya_elongata:1,Zaluzianskya_natalensis:1,Zaluzianskya_pulvinata:1):1):1):1):1,(((((((Watsonia_densifloria:1,Watsonia_lepida:1,Watsonia_paucifolia:1,(Watsonia_wilmsii1:1,Watsonia_wilmsii2:1):1):1,(Lapeirousia_anceps:1,Lapeirousia_dolomitica:1,Lapeirousia_fabricii:1,(Lapeirousia_jacquinii1:1,Lapeirousia_jacquinii2:1):1,Lapeirousia_oreogena:1,Lapeirousia_pyramidalis_regalus:1,Lapeirousia_silenoiedes:1,Lapeirousia_violacea:1):1):1,((Romulea_hantamensis:1,(((Ixia_bellendenii:1,Ixia_panniculata:1,(Ixia_paucifolia1:1,Ixia_paucifolia2:1):1):1,(((Babiana_curviscapa:1,Babiana_dredgei:1):1,(Babiana_ecklonii:1,Babiana_flabellifolia:1):1):1,((((Babiana_framesii1:1,Babiana_framesii2:1):1,Babiana_geniculata:1):1,(Babiana_pubescens:1,Babiana_sambucina_var_longibracteata:1):1):1,(Babiana_sambucina_var_unguiculata:1,Babiana_tubulosa:1):1):1):1):1,((Sparaxis_metelerkampiae:1,(((Tritonia_crispa1:1,Tritonia_crispa2:1):1,(Tritonia_flabellifolia1:1,Tritonia_flabellifolia2:1):1):1,Tritonia_pallida:1):1):1,((((Hesperantha_latifolia:1,Hesperantha_brevicaulis:1):1,(Hesperantha_grandiflora:1,(Hesperantha_scopulosa:1,Hesperantha_woodii:1):1):1):1,(((Geissorhiza_bonaspei1:1,Geissorhiza_bonaspei2:1):1,Geissorhiza_exscapa:1):1,(Geissorhiza_confusa1:1,Geissorhiza_confusa2:1):1):1):1,(Tritoniopsis_revoluta:1):1):1):1):1):1,((Gladiolus_angustus:1,(((Gladiolus_bilineatus:1,Gladiolus_calcaratus:1):1,(Gladiolus_carneus1:1,Gladiolus_carneus2:1):1):1,(Gladiolus_engysiphon:1,(((Gladiolus_floribundus1:1,Gladiolus_floribundus2:1):1,(Gladiolus_macneilii:1,Gladiolus_microcarpus:1):1):1,(Gladiolus_vigilans:1,Gladiolus_virgatus:1):1):1):1):1):1,((Gladiolus_monticola1:1,Gladiolus_monticola2:1):1,Gladiolus_mortonius:1,Gladiolus_oppositiflorus:1,Gladiolus_rhodanthus:1,Gladiolus_undulatus:1,(Gladiolus_varius1:1,Gladiolus_varius2:1):1):1):1):1):1,Nivenia_stenosiphon:1):1,Aristea_spiralis:1):1,((((Disa_cooperi:1,Disa_skullyi:1):1,(Disa_draconis:1,Disa_harveiana:1):1,Disa_nervosa:1,((((Disa_pulchra:1,Disa_oreophila:1):1,Disa_amoena:1):1,Disa_cephalotes:1):1,Disa_ferruginea:1):1):1,(Brownleea_coerulea:1,Brownleea_macroceras:1):1):1,((Aerangis_ellisii1:1,Aerangis_ellisii2:1):1,((Angraecum_arachnites:1,Angraecum_articulata:1,Angraecum_compactum:1,Angraecum_fuscata:1,Angraecum_sesquipedale:1):1,Bonatea_speciosa:1,(Jumellea_teretifiola:1,Neobathiea_grandidierana:1):1):1):1):1):1,Disa_amoena:1):1):1):1);

**Inter-specific insect phylogeny**

(Jadera.haematoloma:1,Curculio.camelliae:1,((((((Moegistorynchus.longirostris1:1,Moegistorynchus.longirostris2:1,Moegistorynchus.longirostris3:1,Moegistorynchus.longirostris4:1,Moegistorynchus.longirostris5:1,Moegistorynchus.longirostris6:1,Moegistorynchus.longirostris7:1,Moegistorynchus.longirostris8:1,Moegistorynchus.longirostris9:1,Moegistorynchus.longirostris10:1):1,(Moegistorynchus.sp.nova1:1,Moegistorynchus.sp.nova2:1):1):1,((Proscoeca.nitidula1:1,Proscoeca.nitidula2:1,Proscoeca.nitidula3:1):1,(Proscoeca.nov.1:1,Proscoeca.nov.2:1,Proscoeca.nov.3:1,Proscoeca.nov.4:1,Proscoeca.nov.5:1,Proscoeca.nov.6:1):1,(Proscoeca.peringueyi1:1,Proscoeca.peringueyi2:1,Proscoeca.peringueyi3:1,Proscoeca.peringueyi4:1,Proscoeca.peringueyi5:1,Proscoeca.peringueyi6:1,Proscoeca.peringueyi7:1,Proscoeca.peringueyi8:1,Proscoeca.peringueyi9:1,Proscoeca.peringueyi10:1,Proscoeca.peringueyi11:1,Proscoeca.peringueyi12:1,Proscoeca.peringueyi13:1,Proscoeca.peringueyi14:1,Proscoeca.peringueyi15:1,Proscoeca.peringueyi16:1,Proscoeca.peringueyi17:1):1,(Prosoeca.ganglbaueri1:1,Prosoeca.ganglbaueri2:1,Prosoeca.ganglbaueri3:1,Prosoeca.ganglbaueri4:1,Prosoeca.ganglbaueri5:1,Prosoeca.ganglbaueri6:1,Prosoeca.ganglbaueri7:1,Prosoeca.ganglbaueri8:1,Prosoeca.ganglbaueri9:1,Prosoeca.ganglbaueri10:1,Prosoeca.ganglbaueri11:1,Prosoeca.ganglbaueri12:1,Prosoeca.ganglbaueri13:1,Prosoeca.ganglbaueri14:1,Prosoeca.ganglbaueri15:1,Prosoeca.ganglbaueri16:1,Prosoeca.ganglbaueri17:1,Prosoeca.ganglbaueri18:1):1,(Prosoeca.longipenis1:1,Prosoeca.longipenis2:1,Prosoeca.longipenis3:1,Prosoeca.longipenis4:1,Prosoeca.longipenis5:1):1,(Prosoeca.robusta1:1,Prosoeca.robusta2:1,Prosoeca.robusta3:1,Prosoeca.robusta4:1):1,((Prosoeca.sp1a:1,Prosoeca.sp1b:1):1,Prosoeca.sp2:1):1):1,((Stenobasipteron.sp1:1,Stenobasipteron.sp2:1):1,(Stenobasipteron.wiedemannii1:1,Stenobasipteron.wiedemannii2:1,Stenobasipteron.wiedemannii3:1,Stenobasipteron.wiedemannii4:1,Stenobasipteron.wiedemannii5:1):1):1):1,((Philoliche.aethiopica1:1,Philoliche.aethiopica2:1,Philoliche.aethiopica3:1,Philoliche.aethiopica4:1):1,(Philoliche.gulosa1:1,Philoliche.gulosa2:1,Philoliche.gulosa3:1,Philoliche.gulosa4:1,Philoliche.gulosa5:1,Philoliche.gulosa6:1,Philoliche.gulosa7:1,Philoliche.gulosa8:1,Philoliche.gulosa9:1,Philoliche.gulosa10:1,Philoliche.gulosa11:1):1,(Philoliche.rostrata1:1,Philoliche.rostrata2:1,Philoliche.rostrata3:1,Philoliche.rostrata4:1,Philoliche.rostrata5:1,Philoliche.rostrata6:1,Philoliche.rostrata7:1,Philoliche.rostrata8:1,Philoliche.rostrata9:1,Philoliche.rostrata10:1,Philoliche.rostrata11:1,Philoliche.rostrata12:1,Philoliche.rostrata13:1,Philoliche.rostrata14:1,Philoliche.rostrata15:1,Philoliche.rostrata16:1):1,Tabanid.sp.:1):1):1,((Meneris.tulbaghia1:1,Meneris.tulbaghia2:1):1,(((((Manduca.quinquemaculata1:1,Manduca.quinquemaculata2:1,Manduca.quinquemaculata3:1,Manduca.quinquemaculata4:1,Manduca.quinquemaculata5:1):1,Manduca.rustica:1):1,Xanthopan.morgani.praedicta:1):1,Agrius.convolvuli:1):1,Basiotha.schenki:1,(Hippotion.celerio1:1,Hippotion.celerio2:1,Hippotion.celerio3:1):1,(Hyles.lineata1:1,Hyles.lineata2:1):1,(Panogena.lingens1:1,Panogena.lingens2:1,Panogena.lingens3:1,Panogena.lingens4:1,Panogena.lingens5:1,Panogena.lingens6:1,Panogena.lingens7:1):1,Sphinx.vashti:1,Thereta.capensis:1):1):1):1,((Rediviva.neliana1:1,Rediviva.neliana2:1):1,Rediviva.pallidulaca.ganglbaueri1:1):1):1);

End;
